# Fatal Dog Attacks in Italy (2009–2025): The Urgent Need for a National Risk Registry

**DOI:** 10.3390/ani15243523

**Published:** 2025-12-06

**Authors:** Fabrizio Iarussi, Francesco Sessa, Serena Piccirillo, Martina Francaviglia, Alessandra Recchia, Antonella Colella, Matteo Bolcato, Monica Salerno, Angelo Peli, Cristoforo Pomara

**Affiliations:** 1 Department of Precision and Regenerative Medicine and Ionian Area, Section of Veterinary Clinics and Animal Production, University of Bari Aldo Moro, S.P. Valenzano-Casamassima Km 3, 70010 Valenzano, BA, Italy; alessandra.recchia1@uniba.it (A.R.); antonella.colella@uniba.it (A.C.); 2 Department of Medical, Surgical and Advanced Technologies “G.F. Ingrassia”, University of Catania, 95121 Catania, CT, Italy; francesco.sessa@unict.it (F.S.); serena.piccirillo@hotmail.it (S.P.); martinafrancaviglia95@gmail.com (M.F.); monica.salerno@unict.it (M.S.); cristoforo.pomara@unict.it (C.P.); 3 Department of Medicine, Saint Camillus International University of Health and Medical Sciences, 00131 Rome, RM, Italy; matteo.bolcato@unicamillus.org; 4 Department of Life Quality Studies, University of Bologna, 40127 Bologna, BO, Italy; angelo.peli@unibo.it

**Keywords:** fatal dog attacks, canine aggression, mauling, public health surveillance, dog attack epidemiology, national risk registry

## Abstract

Fatal dog attacks are rare but devastating events with significant public health, forensic, and legal implications. Italy lacks a centralized registry for such incidents, limiting the ability to monitor trends and implement preventive strategies. This retrospective study analyzes all verified fatal dog attacks in Italy from 2009 to 2025. Data were collected from national and regional media, cross-verified, and organized into a comprehensive database. Descriptive statistics, chi-square tests, logistic and Poisson regressions, and interaction analyses were performed to identify patterns in victim demographics, breed involvement, ownership status, and environmental context. A total of 54 fatal attacks were recorded, with an increasing trend observed in the last five years. Elderly individuals (≥65) and preschool-aged children (≤4) were the most affected groups. Molosser and bull-type breeds were implicated in 69% of cases, and 92.6% of attacks involved owned dogs—more than half belonging to the victim. Private settings accounted for 66.7% of incidents. Comparative analysis with U.S. data revealed similar demographic and breed-related patterns, but also highlighted Italy’s lack of a centralized behavioral risk registry. Fatal dog attacks in Italy follow recurring and preventable patterns. The absence of a national database severely limits surveillance and intervention. A centralized behavioral risk registry, modeled on international systems, should be established to support early detection, policy development, and multidisciplinary collaboration.

## Simple Summary

Fatal attacks by dogs are rare but deeply tragic events that affect families and communities. This study examined every confirmed case of a person killed by a dog in Italy from 2009 to 2025. The aim was to understand who is most at risk, which types of dogs are usually involved, and under what circumstances these events occur. The analysis revealed that very young children and elderly individuals were the most frequent victims, and that most incidents occurred in private settings such as homes or gardens. In almost all cases, the attacking dogs were owned pets, often belonging to the victim’s household. Large and powerful breeds, such as molossers and bull-type dogs, appeared most frequently in the fatal cases. These findings show that fatal dog attacks typically occur in predictable contexts and involve preventable situations, especially when adequate supervision or responsible ownership is lacking. The absence of a national system to record and monitor such events makes it difficult to intervene early or prevent recurrence. Establishing a national registry for behavioral risk could help protect both people and animals through improved awareness, education, and timely intervention.

## 1. Introduction

Fatal dog attacks represent a serious concern for public health, forensic medicine, and legal policy. Globally, dog bites are among the most common animal-related injuries, with the United States reporting approximately 4.5 million incidents annually, including 885,000 requiring medical attention and an average of 20 fatalities per year. While the United States benefits from publicly accessible compilations such as DogsBite.org, the European context and Italy remain underrepresented in systematic data collection and analysis [1]. Although DogsBite.org is not a scientific or peer-reviewed database, it is a publicly accessible archive that compiles fatal dog attacks in the United States, and in this study, it is used solely as a descriptive point of comparison rather than as a source of epidemiological evidence. A systematic review by Giovannini et al. (2023) [2] examined the medico-legal implications of canine aggression on an international scale. In Italy, the only national epidemiological study on fatal dog attacks was conducted by Ciceroni and Gostinicchi, covering the period from 1984 to 2009 [3]. Their work provided a foundational profile of victims, breeds involved, and environmental contexts, but left a significant gap in longitudinal surveillance. Since then, Italian literature has focused primarily on ethological, behavioral, and forensic aspects [4], without updating the epidemiological framework.

In response to rising public concern, the Italian Ministry of Health in 2003 issued an ordinance (the so-called Sirchia Ordinance), renewed in 2004, which imposed breed-specific restrictions and mandated the use of leashes and muzzles for dogs deemed dangerous [5]. However, this approach was replaced in 2009 by a new ordinance emphasizing owner accountability and behavioral risk reporting, eliminating the breed list altogether [6]. Despite these regulatory shifts, no national registry or centralized database has been established to monitor fatal dog attacks or behavioral risk factors.

Recent forensic studies have highlighted the complexity of canine aggression. Pomara et al. (2011) [7] demonstrated the utility of bite mark analysis in pack attacks, and more recently, Giovannini et al. (2025) [8] expanded this approach through a morphometric study that improved the reliability of lesion-to-weapon correlations and the interpretation of bite mark patterns in forensic investigations.

Iarussi et al. (2020) [9] proposed a novel forensic method for identifying aggressive dogs through human DNA traces detected in the oral cavity. Benevento et al. (2021) further emphasized the need for multidisciplinary collaboration between forensic medicine and veterinary science [10]. In this context, Roccaro et al. (2021) provided an additional perspective by applying forensic genetics to identify the species or individual animal responsible for an attack, demonstrating the potential of molecular methods in complex medico-legal cases [11]. Giovannini et al. (2023) [2] conducted a systematic review of more than 100 studies on the medico-legal implications of dog bites, underscoring the fragmented nature of current research and the absence of comprehensive epidemiological data.

In contrast, the United States maintains a publicly accessible database that tracks fatal dog attacks across multiple variables, including breed, ownership, victim age, and environmental context, enabling real-time surveillance and policy evaluation. Between 2020 and 2023, DogsBite.org recorded an average of 55–63 fatalities annually, with pit bulls involved in over 60% of multi-dog attacks and children under 9 and adults over 30 representing the majority of victims [12]. This model offers a valuable reference for Italy, where the lack of centralized data continues to hinder effective prevention and intervention strategies.

Given this context, the present study aims to fill the epidemiological gap by providing a systematic and updated analysis of fatal dog attacks in Italy from 2009 to 2025. Building upon the methodology of Ciceroni and Gostinicchi [3], this research extends the observation window and incorporates multivariate statistical analyses to identify trends in victim demographics, breed involvement, ownership status, and environmental settings. The ultimate goal is to inform public health policy, enhance forensic protocols, and advocate for the creation of a national behavioral risk registry modeled on international best practices.

## 2. Materials and Methods

### 2.1. Data Collection and Media Search Methodology

This study examined fatal dog attacks in Italy during the period 2009–2025 by systematically collecting information from verified journalistic sources. A manual, year-by-year search was performed using the digital archives of major national newspapers, including La Repubblica, Corriere della Sera, Il Messaggero, La Stampa, Il Mattino, and La Gazzetta del Mezzogiorno, as well as local outlets covering the specific areas where the attacks occurred. Google News was also consulted to supplement this search and identify additional regional reports not captured through the primary newspaper archives.

The search employed Italian-language keywords commonly used in national and regional media reports of fatal dog attacks (e.g., “ucciso da cane”, “sbranato”, “morso cane”, “aggressione mortale”, “bambino ucciso”, “anziano ucciso”). These keywords were selected to reflect authentic journalistic phrasing and to ensure that the search procedure could be replicated. Only cases explicitly reporting a fatal outcome were retained; non-fatal or “near-fatal” events were excluded because they are inconsistently documented and cannot be independently verified from media sources.

Each fatal event was validated through at least two independent reports. For every confirmed case, the following data were extracted: date, region, victim demographics, ownership status, dog breed or type (when reported), and contextual information about the attack. Fatalities were classified as either death on scene or death in hospital, depending on whether the victim died immediately or subsequently due to injuries sustained in the attack.

The 54 confirmed fatal cases identified through this procedure are listed in Table 1, together with the main verified media source and the classification of the fatal outcome (death on scene vs. death in hospital). Detailed case-level variables are reported in Supplementary Table S1; the complete data dictionary defining all variables is provided in Supplementary Table S2, and full incident narratives with links to all primary articles are presented in File S1.

In Italy, official mortality statistics coded according to ICD-10 are not publicly accessible at a level of detail that would allow deaths specifically attributable to incidents classified under W54 (“bitten or struck by dog”) to be identified and analyzed. This limitation has been formally acknowledged by the Italian Ministry of Health, which in 2024 issued a national request for standardized regional reporting of dog bites and aggressive incidents (“Nota per la rendicontazione regionale degli episodi di morsicatura/aggressione”, Ministry of Health, 2024) [13]. Accordingly, media-based case identification currently represents the only practicable approach for compiling a nationwide series of fatal dog attacks.

### 2.2. Definition of Study Variables

For each incident included in the analysis, data were collected on key epidemiological variables. These included the date (year and month) and location (province) of the event, as well as demographic details of the victims, such as age, sex, and number. Information was also gathered on the dogs involved, including their number, breed (or morphological/functional type), and ownership status, whether the animals belonged to the victim, to third parties, or were free-roaming or stray.

Particular attention was paid to the number of dogs involved in each event. Because news reports varied considerably in how they described the animals present, attacks were classified descriptively as single-dog, dyadic (two-dog), or larger multi-dog events. This terminology was adopted solely for operational clarity and does not imply any ethological threshold or behavioral interpretation.

The environmental context in which the attack occurred was categorized by distinguishing between domestic settings, private gardens, public urban areas, and rural spaces. The database structure was designed to mirror that of the earlier study by Ciceroni and Gostinicchi (2009) [3], thereby enabling direct comparisons with the cases documented between 1984 and 2009.

### 2.3. Data Analysis and Bias Control Strategies

The data collected were compiled into a structured database designed for both descriptive and exploratory statistical analysis, with the objective of identifying temporal trends, recurring characteristics, and potential risk factors associated with fatal dog attacks.

To ensure methodological rigor and minimize interpretative bias, several control strategies were implemented. Only incidents confirmed by at least two independent journalistic sources were included in the dataset. All available information was cross-verified to ensure narrative consistency and data reliability.

### 2.4. Statistical Analysis

Frequencies and proportions were calculated for categorical variables. Measures of central tendency (mean and median) and dispersion (standard deviation and interquartile range) were computed for continuous variables such as victim age. Visualizations included bar charts, heatmaps, and stacked plots. Inferential analyses included chi-square tests to assess independence and goodness-of-fit across sex, age groups, geographic regions, and ownership categories. Binary logistic regression modeled the likelihood of attacks occurring in private vs. public settings, using predictors such as victim age, breed type, and ownership status. Multinomial logistic regression was used to explore associations between victim age groups and dog ownership categories.

To assess temporal trends in annual fatality counts, we fitted a Poisson regression model with year as a continuous predictor. The appropriateness of the Poisson model was evaluated by calculating the Pearson chi-square to degrees-of-freedom ratio to check for overdispersion. In addition, a negative binomial model with the same structure was fitted for comparison, and its information criteria (AIC and BIC) were examined to determine which model provided the best overall fit. Interaction effects were tested between age and environment, and between breed type and ownership, to identify compound risk factors. Model diagnostics included Hosmer–Lemeshow tests, AIC/BIC comparisons, and residual analysis. All statistical procedures were conducted using JASP (v0.18.3) and R (v4.3.1), with supplementary processing in Microsoft Excel.

Sensitivity analysis. Given the potential for incomplete media reporting in the most recent years, a sensitivity analysis was performed by repeating the Poisson regression after removing the last two years of data (2024–2025). The structure of the model was kept identical to the primary analysis, with “year” entered as a continuous predictor of annual fatality counts. This approach allowed us to verify whether possible under-reporting in the most recent period could affect the interpretation of the temporal trend.

## 3. Results

### 3.1. Annual Incidence of Fatal Attacks

Between 2009 and 2025, a total of 54 fatal dog attacks were recorded in Italy. Annual incidence ranged from 1 to 5 cases per year, with peaks in 2012, 2024, and 2025. The distribution of fatal incidents varied over time, with marked peaks in 2012, 2024, and 2025 (five deaths each) and a minimum in 2014 (one death).

**Figure 1:**
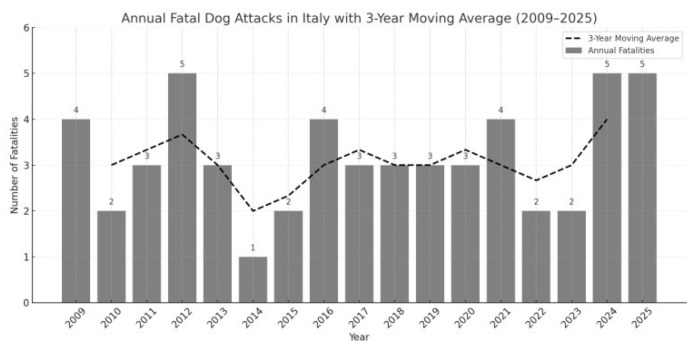
Annual number of human fatalities resulting from fatal dog attacks in Italy from 2009 to 2025 (*n* = 54). Although a few peaks are observed (2012, 2024, 2025), the overall trend remains relatively stable throughout the study period.

Although year-to-year fluctuations were observed, the overall number of fatalities showed no consistent upward or downward trend. This pattern is more clearly visualized using a 3-year moving average, which smooths short-term variations and highlights broader temporal dynamics (Figure 1).

Poisson regression analysis showed no statistically significant upward trend over time (Incidence Rate Ratio [IRR] = 1.03; 95% CI: 0.98–1.08; *p* = 0.24), suggesting relative temporal stability. The regression coefficient for year was also non-significant when estimated directly (β = 0.0147; *p* = 0.598). The dispersion test indicated no overdispersion (Pearson chi-square/df = 0.45), confirming that a Poisson model was appropriate. A model comparison showed that the negative binomial alternative had substantially higher information criteria (AIC = 82.10; BIC = 83.77) than the Poisson model (AIC = 61.74; BIC = 63.40), supporting the choice of the Poisson specification.

Sensitivity analysis showed that excluding the years 2024–2025 did not alter the interpretation of the temporal trend. The annual counts for 2009–2023 (mean = 3.2 events/year) showed the same irregular oscillation observed in the full dataset, with no indication of a systematic upward or downward trajectory. The removal of the final two years therefore did not modify the overall conclusion that no statistically detectable temporal trend was present across the study period.

### 3.2. Geographic Distribution of Fatal Attacks

Fatal attacks were distributed across macro-regions as follows: South (40.7%), North (31.5%), and Center (27.8%). A chi-square test revealed no significant deviation from a uniform distribution (χ^2^ = 1.44; *p* = 0.49). When adjusted for regional dog population density, Southern Italy showed a slightly elevated risk ratio (RR = 1.21), although this was not statistically significant. To adjust the regional risk ratio for dog population density, official data on registered dogs were obtained from the National Canine Registry maintained by the Italian Ministry of Health [14]. These figures were supplemented with territorial estimates published by Legambiente [15].

**Figure 2:**
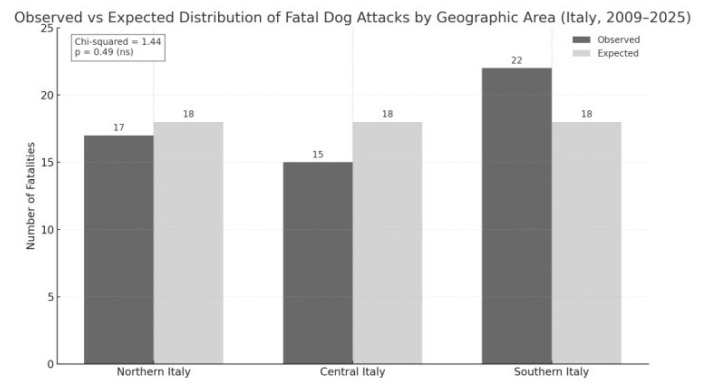
Geographic distribution of human fatalities resulting from fatal dog attacks in Italy between 2009 and 2025. Bars show observed and expected counts for each macro-region. The chi-squared test (χ^2^ = 1.44; *p* = 0.49) revealed no statistically significant difference.

Macro-regional classification (North, Center, South) followed the standard ISTAT framework. The aggregated dog population data were used as denominators to calculate region-specific risk ratios, allowing for a more accurate epidemiological interpretation of the distribution of fatal dog attacks across Italy. These findings suggest that the observed variation may be attributable to random fluctuations rather than true regional predispositions (Figure 2).

### 3.3. Victim Distribution by Sex

**Figure 3:**
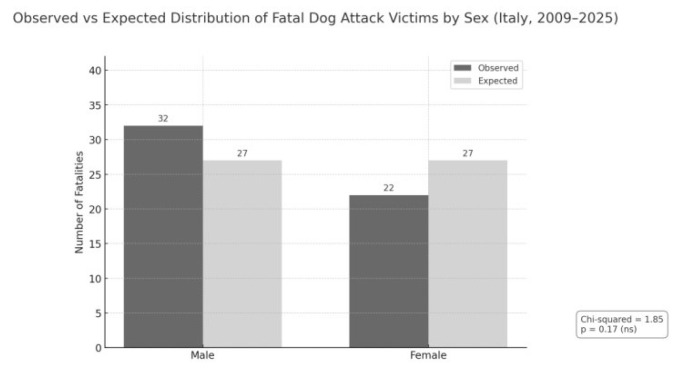
Observed versus expected number of fatalities from dog attacks by sex in Italy (2009–2025). The slight predominance of male victims was not statistically significant, as indicated by the chi-squared test (χ^2^ = 1.85; *p* = 0.17).

Among the 54 documented fatalities between 2009 and 2025, 32 were male (59.3%) and 22 were female (40.7%). Although a higher number of male victims was observed, the difference did not reach statistical significance when tested against a uniform distribution (χ^2^ = 1.85; *p* = 0.17). These findings suggest that fatal dog attacks affect both sexes with comparable frequency, without evidence of a strong sex-based predilection (Figure 3).

### 3.4. Victim Distribution by Age Group

**Figure 4:**
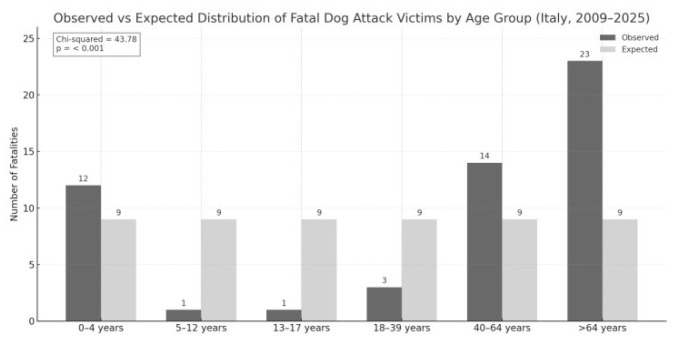
Age-based distribution of human fatalities caused by dog attacks in Italy between 2009 and 2025. The analysis shows that both elderly individuals and young children experienced disproportionately high fatality counts compared to other age groups, as supported by a significant chi-squared result (χ^2^ = 43.78; *p* < 0.001).

Analysis of victim age revealed a markedly uneven distribution across the six age groups. Individuals over 64 years of age represented the most affected category, with 23 fatalities (42.6%), followed by adults aged 40–64 years, who accounted for 14 cases (25.9%). Preschool-aged children (0–4 years) constituted the third most affected group, with 12 victims (22.2%). In contrast, school-aged children (5–12 years) and adolescents (13–17 years) each recorded only 1 fatality (1.9%), while young adults aged 18–39 accounted for 3 cases (5.6%). The chi-square test confirmed a statistically significant deviation from a uniform distribution (χ^2^ = 43.78; *p* < 0.001), indicating increased vulnerability among the youngest and oldest individuals (Figure 4).

### 3.5. Breed Types Involved in Fatal Attacks

**Figure 5:**
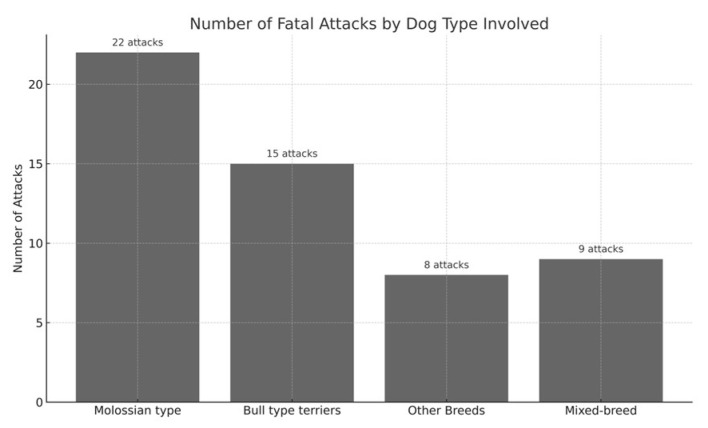
Bar chart showing the frequency of fatal dog attacks in Italy by general dog type. Molossian-type type were involved in the highest proportion of incidents (41%), followed by bull-type terriers (28%), mixed-breed dogs (17%), and other purebred dogs (15%).

**Figure 6:**
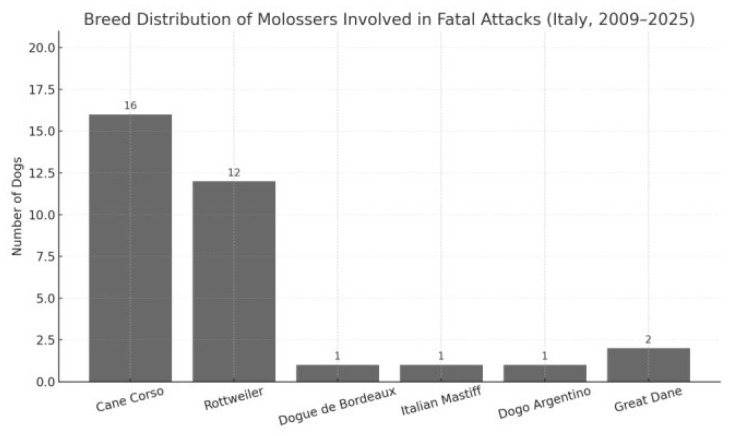
Detailed breakdown of molosser breeds involved in fatal dog attacks. Cane Corso and Rottweiler were the most represented.

A total of 54 fatal events were recorded during the study period. Because several attacks involved more than one dog, the number of individual dogs involved (*n* = 83) was higher than the number of events. To avoid ambiguity, all numbers in this section distinguish between events (cases) and individual dogs. Full case-by-case details, including the number of dogs involved in each event and their respective types or breeds, are reported in Table S1. Across the 54 events, four major dog-type categories were identified: molossian-type dogs, bull-type terriers, other purebred breeds, and mixed-breed dogs. Their overall distribution is illustrated in Figure 5. Molossian-type dogs were involved in 22 events (40.7%), accounting for 33 individual dogs. The breeds most frequently represented within this category were Cane Corso and Rottweiler, followed by isolated instances of Dogue de Bordeaux, Italian Mastiff, Dogo Argentino, and Great Dane, as shown in Figure 6.

**Figure 7:**
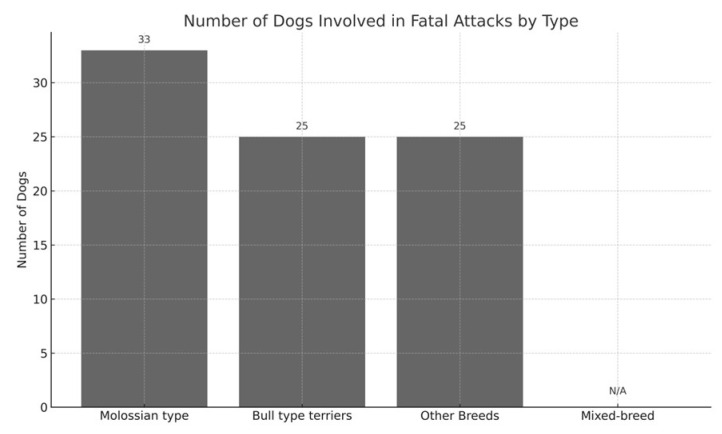
Stacked bar chart comparing the total number of individual dogs implicated in fatal attacks, grouped by general dog type.

Bull type terriers were involved in 15 events (27.8%), with a total of 25 individual dogs. Several incidents in this group included two or more dogs acting together. A comparative visualization of the number of individual dogs implicated across dog-type categories is provided in Figure 7.

Other purebred breeds were implicated in 8 events (14.8%), involving 25 individual dogs. These included German Shepherds, Belgian Malinois, Maremmano–Abruzzese Sheepdogs, and Czechoslovakian Wolfdogs. In several of these events, multiple dogs of the same breed were involved.

Mixed-breed dogs were involved in 9 events (16.7%). For this category, the number of individual dogs could not always be reliably determined, as many journalistic sources simply described the attackers as a “pack of stray dogs” without specifying the exact number of animals.

Taken together, at least 83 identifiable dogs participated in the 54 fatal events, in addition to an undetermined number of mixed-breed dogs.

According to the FCI classification system, the breeds most frequently involved in fatal attacks belonged to three main groups: Group 2 Section 2 (molossian type), Group 3 Section 3 (Bull-type terriers), and Group 1 Section 1 (Sheepdogs) [16]. This grouping was used to support the interpretation of breed-related patterns observed in this study.

### 3.6. Ownership Status of Attacking Dogs

**Figure 8:**
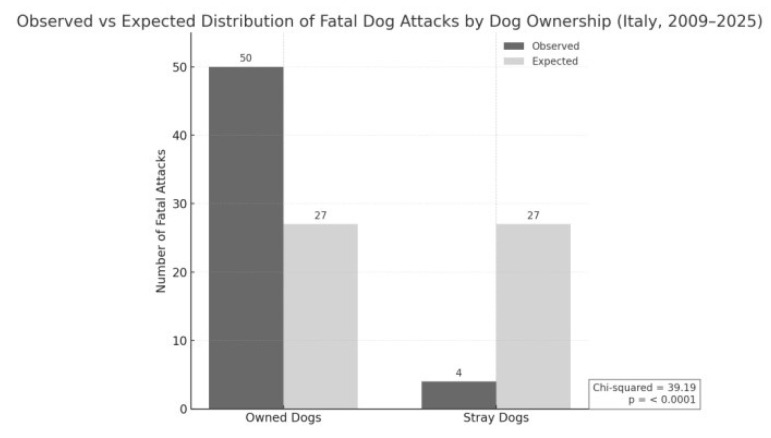
The vast majority of fatal attacks between 2009 and 2025 were committed by owned dogs (*n* = 50), while only a small fraction involved stray dogs (*n* = 4).

Analysis of dog ownership status revealed that the overwhelming majority of fatal attacks in Italy between 2009 and 2025 were perpetrated by owned dogs (*n* = 50; 92.6%), while only 4 cases (7.4%) involved stray dogs. When compared with an expected even distribution, the observed frequencies differed significantly, as confirmed by a chi-squared test (χ^2^ = 39.19; *p* < 0.0001), indicating a marked overrepresentation of attacks by owned dogs (Figure 8).

**Figure 9:**
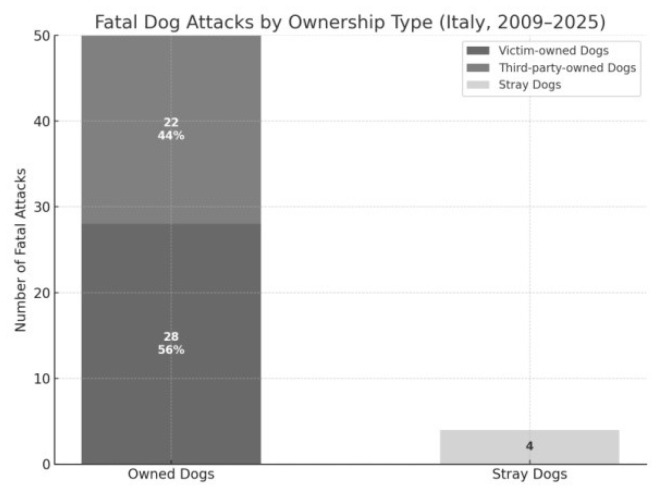
Among the 50 fatal attacks involving owned dogs, 56% were perpetrated by dogs owned by the victim, and 44% by dogs belonging to third parties.

Among the 50 attacks involving owned dogs, further classification showed that 28 cases (56%) were caused by dogs owned by the victim, whereas 22 cases (44%) involved dogs owned by third parties. This distribution was also statistically significant (χ^2^ = 17.33; *p* = 0.00017), suggesting that attacks by dogs belonging to the victim were notably more frequent than might be expected by chance (Figure 9).

### 3.7. Contextual Setting of Fatal Dog Attacks

**Figure 10:**
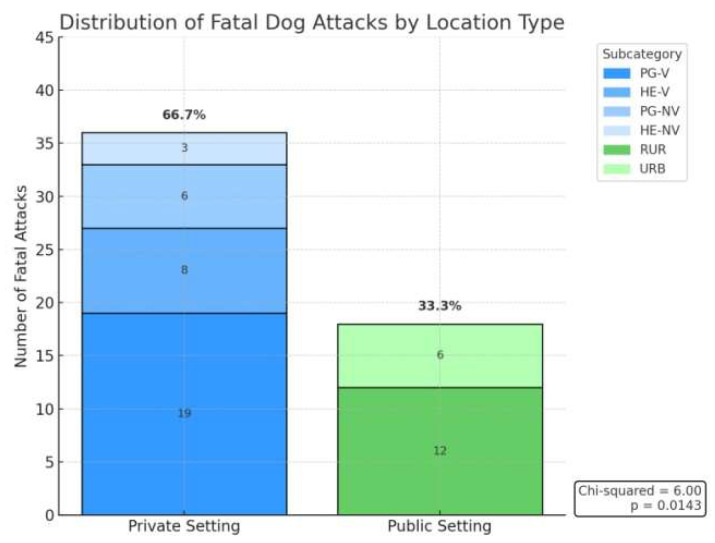
Distribution of fatal dog attacks across private and public settings, highlighting a higher concentration of events within privately controlled environments. Legend: HE-NV = Non-victim home environment; PG-V = Victim’s private garden; PG-NV = Non-victim private garden; RUR = Rural environment; URB = Urban environment.

The analysis of the setting in which fatal dog attacks occurred reveals a predominance of incidents in private spaces. Of the 54 documented cases, 36 (66.7%) occurred in private settings, including the victim’s own garden (*n* = 19) or home (*n* = 8), as well as gardens (*n* = 6) and homes (*n* = 3) belonging to others. Public settings accounted for the remaining 18 cases (33.3%), with rural environments (*n* = 12) being more frequently involved than urban areas (*n* = 6). Statistical testing confirmed a significantly higher occurrence of fatal attacks in private settings compared to what would be expected under a uniform distribution (χ^2^ = 6.00; *p* = 0.0143) (Figure 10).

### 3.8. Multivariate Analysis

#### 3.8.1. Age Group vs. Setting

**Figure 11:**
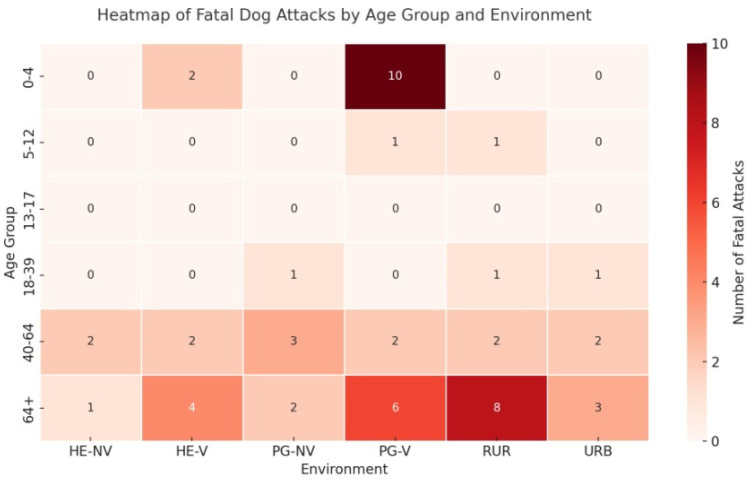
Heatmap of fatal dog attacks by victim age group and environment. Children (0–4 years) were mainly attacked in their own gardens, while elderly victims (≥65) were most often involved in rural settings. Adults (40–64) showed a more varied distribution across environments.

Cross-analysis between victim age group and environment of the attack reveals distinctive distribution patterns. Preschool-aged children (0–4 years) were primarily involved in incidents occurring in the victim’s private garden (PG-V), while elderly victims (>64 years) were most frequently attacked in rural environments (RUR). Adults aged 40–64 years showed a more heterogeneous distribution, with cases occurring across home environments, private gardens, and open spaces. These findings suggest a potential relationship between age-related vulnerability and the environmental context of fatal attacks, with important implications for surveillance, risk management, and prevention strategies (Figure 11).

#### 3.8.2. Victim Age vs. Ownership of Attacking Dog

**Figure 12:**
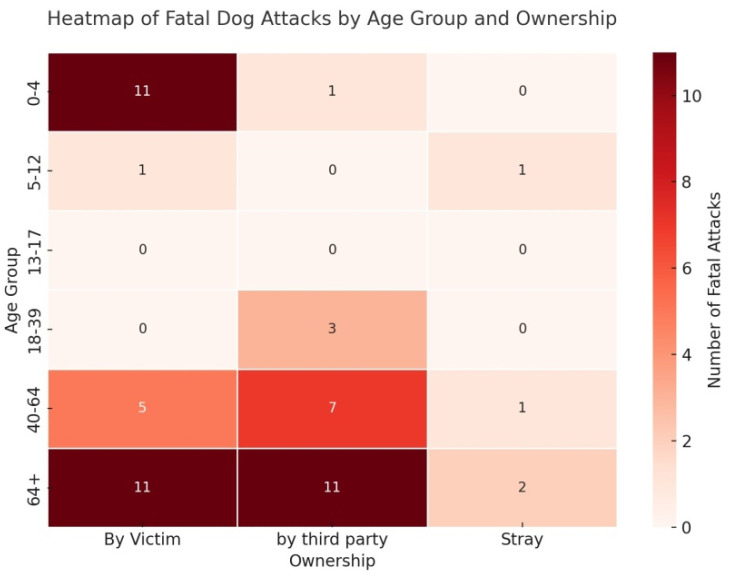
Heatmap of fatal dog attacks in Italy (2009–2025) by victim age group and dog ownership. Children (0–4 years) were attacked almost exclusively by dogs from their own household, adults (18–39) by dogs owned by third parties, and the elderly (≥65) mainly by dogs of third parties. Stray dogs were rarely involved.

Cross-analysis between victim age group and the ownership status of the attacking dog highlights several notable patterns. Among children aged 0–4 years, fatal attacks occurred almost exclusively with dogs owned by the victim’s household (6 out of 7 cases), indicating that domestic and familial contexts are the primary risk environments for this age group. In contrast, victims aged 18–39 years were attacked exclusively by dogs owned by third parties. In older age groups, particularly those over 64, a high frequency of attacks involved dogs owned by third parties (8 cases), followed by dogs owned by the victim (5 cases). Attacks by stray dogs were very rare, with only two such cases recorded in adult and elderly victims (Figure 12).

## 4. Discussion

The present study offers a unique opportunity to examine the phenomenon of fatal dog attacks in Italy across two distinct timeframes governed by different regulatory strategies. The first period (1984–2009) was characterized by breed-specific preventive measures, while the second (2009–2025) marked a shift toward owner accountability and the behavioral assessment of individual animals [3,5,6]. Despite this regulatory evolution, the annual number of fatal attacks increased from 1.28 to 3.18 cases per year, with a notable rise observed in the most recent five-year interval. These findings raise concerns about the practical effectiveness of a prevention model based solely on individual responsibility.

The increase in fatal incidents is likely driven by multiple contributing factors rather than a single cause. According to data from the National Canine Registry, the dog population in Italy has more than doubled over the past decade [14,15]. It is therefore plausible that the rising frequency of human–dog interactions has contributed to a higher absolute risk of fatal attacks. However, this interpretation should be considered alongside additional behavioral, management, and environmental determinants emerging from detailed case analyses.

From an environmental perspective, 66.7 percent of attacks occurred in private settings, particularly within the victim’s home or garden. Although this could suggest a shift toward closer domestic cohabitation, comparison with the 1984–2009 data reveals that private spaces have consistently represented the primary context of fatal attacks, suggesting a structural pattern rather than a recent trend.

The analysis of victim demographics confirms heightened vulnerability at both ends of the age spectrum. Children aged 0–4 years and elderly individuals (≥64 years) were the most frequently affected groups. The heatmap analysis offers important insights: among young children, 91.7 percent of fatalities involved dogs owned by the victim’s household, and 10 of the 12 deaths occurred in the family’s private garden (PG-V) (Figure 11 and Figure 12). These findings clearly indicate that the risk does not arise from mere cohabitation with potentially dangerous dogs, but rather from the absence or inadequacy of adult supervision in domestic settings. Leaving very young children unattended in environments shared with animals capable of causing severe injury constitutes a serious and often underestimated hazard. This issue also carries legal implications: under Italian criminal law, specifically the Italian Penal Code (Articles 570 and 571), failure to adequately supervise minors, especially when it results in injury or death, may constitute criminal negligence. In this context, the lack of supervision in the presence of potentially dangerous dogs entails not only parental responsibility but also legal liability.

Fatal attacks involving infants require separate consideration, as their circumstances and injury patterns differ markedly from those of older children. In our dataset of twelve infant victims (0–4 years), ten were positioned on the floor or at ground level at the moment of the attack, while two were being held in the arms of an adult caregiver. Five incidents occurred while the child had been temporarily entrusted to relatives, whereas in seven cases at least one parent was present in the household. These findings indicate that close adult proximity alone does not reliably mitigate risk, and that the motivational forces underlying these events can be substantial. The fact that two infants were attacked while being held in an adult’s arms further suggests that escalation can occur despite direct supervision.

Published forensic case reports describe repeated, deep and often craniofacial injuries in fatal attacks on infants, consistent with predatory motor sequences rather than defensive or territorial aggression [17,18]. Infants possess several characteristics that may act as potent prey-like stimuli, including small body size, limited motor coordination and high-pitched vocalizations. These features can facilitate rapid escalation of attacks in familiar environments, often with minimal or no preceding warning behavior [19]. Additional behavioral analyses indicate that predatory sequences in dogs may involve a silent, direct approach, gripping and lack of volitional release, patterns well documented in evaluations of predatory and offensive aggression [20]. Observational evidence further shows that dogs with high reactivity or heightened arousal may attack without vocalization and may escalate suddenly even in the absence of provocation [21]. Ethological literature also describes predatory behavior in dogs as an instinctive, internally reinforcing sequence not dependent on hunger [22], which may help explain why several attacks progressed despite the presence of adults. Together, these findings support the interpretation that small, immobile children can elicit a predatory response in dogs exhibiting high reactivity or reduced behavioral inhibition [21].

Overall, the combined evidence suggests that fatal attacks on infants represent a distinct risk scenario shaped by predictable vulnerability and by the activation of predatory motivational systems in the dog. This highlights the need for stringent physical separation between infants and dogs with high predatory or offensive potential, even in domestic settings. A synthesis of the principal trigger-related factors observed in the 12 infant fatalities is provided in Table 2.

A similar dynamic of age-related vulnerability emerges in older adults, who accounted for 23 fatalities in our dataset. Previous research shows that elderly individuals often exhibit reduced muscle strength, slower reaction times and impaired balance, all of which diminish their ability to interrupt or avoid a dog attack once it begins [23]. Medico-legal studies further indicate that older victims tend to sustain more severe injuries to sensitive anatomical regions, reflecting their limited defensive capacity in the face of sudden aggression [2]. Additional work on human–animal interactions in later life highlights that older adults may encounter specific risks in domestic settings, where physically powerful dogs may behave unpredictably despite familiarity [24]. From an ethological perspective, dogs may also react more forcefully toward individuals who display physical weakness or instability, a behavioral pattern described in analyses of predatory and offensive responses in domestic dogs [25].

News reports seldom provide medical detail, yet several cases in our dataset include journalistic descriptions suggesting additional fragility beyond age alone. These accounts, available through the source links provided in Supplementary File S1, refer to the possibility of a medical event preceding the attack (cases 7, 24, 26, 53 and 51), a fall immediately before the aggression (cases 17, 26, 49), or documented disability or chronic illness (cases 1, 24, 49). While such information cannot be clinically verified, it illustrates how pre-existing frailty or acute incapacity may have intersected with the dynamics of the fatal event. The overrepresentation of elderly victims, together with these narrative examples, aligns closely with current scientific understanding of how age-related physical decline contributes to severe outcomes in dog attacks.

Among the elderly, the pattern is more heterogeneous. Fatalities were distributed across multiple environments—including private homes (HE-V), gardens (PG-V, PG-NV), and rural settings and involved dogs owned both by the victim and by third parties, as well as strays. This complexity may reflect the reduced ability of older individuals to respond to aggression or escape once an attack is initiated, irrespective of the context.

Beyond individual vulnerability, a separate consideration concerns the dynamics of attacks involving more than one dog. In our dataset, 19 incidents (35.2 percent) were perpetrated by a single dog, 15 (27.8 percent) by a dyad, and 20 events (37.0 percent) involved three or more dogs. Although the behavioral detail provided by journalistic sources was often limited, these figures confirm that multi-dog aggression, whether involving two dogs or larger groups, represents a substantial proportion of fatal events. Experimental and forensic observations indicate that arousal and aggressive motivation in dogs can be socially facilitated, with the behavior of one dog lowering the inhibitory threshold of another and contributing to more sustained or severe attacks [26]. Even dyadic events may therefore constitute a meaningful form of multi-dog aggression, since mutually reinforcing actions can already emerge when two dogs act together [27]. Studies of free-ranging dogs and forensic reconstructions of fatal incidents further show that larger groups may display more complex forms of coordination, including simultaneous targeting of different body regions, prolonged pursuit and reduced responsiveness to human intervention [28,29]. While the level of detail in news reports does not permit a fine-grained reconstruction of interactional dynamics for individual cases, acknowledging the role of socially facilitated aggression is important for understanding why multi-dog events, whether involving two animals or larger packs, may escalate rapidly and produce particularly severe outcomes.

While no fatal event in our dataset could be clearly classified as redirected aggression, two incidents involved a possible preceding interaction with another animal, namely a cat in case 17 and the presence of a small dog in case 24 (Supplementary File S1). The journalistic sources did not provide sufficient behavioral detail to determine whether redirection played a role, but these situations illustrate how high arousal during inter-animal interactions may precede sudden escalation. Redirected aggression remains a recognized behavioral pathway that can result in severe human injury. In such situations, intense arousal during inter-dog aggression may be redirected toward a nearby human, particularly when the person attempts to separate the animals or protect their own dog. This mechanism, well described in the behavioral literature [30], is relevant to prevention strategies in households managing dogs with a history of dog-directed aggression, even if it was not confirmed among the cases included in the present study.

Journalistic reports seldom provided detailed information on everyday husbandry, yet a subset of cases contained explicit descriptions of the conditions in which the attacking dogs were kept. At least three incidents involved dogs maintained on a tether, and at least five cases described animals confined within enclosures or restricted spaces. In addition, in at least four fatal events the dogs were identified as property or livestock guardians rather than companion animals. While these figures represent only a conservative fraction of the dataset, they nonetheless point to management patterns that may influence welfare and behavioral stability. Experimental evidence shows that prolonged tethering can compromise welfare and heighten behavioral reactivity in dogs [31]. Similarly, livestock-guarding dogs raised with limited human contact exhibit a significantly higher likelihood of human-directed aggression compared to dogs reared in mixed or family environments, highlighting the relevance of early social exposure [32]. More broadly, welfare research on working dogs indicates that environmental restriction, insufficient stimulation and suboptimal housing conditions can impair behavioral regulation and increase stress responses [33]. Within this framework, even the limited management details recoverable from journalistic accounts suggest that tethering, confinement and traditional guarding roles may represent contextual factors that contribute to the escalation of some fatal attacks.

A smaller set of incidents in our dataset appeared, based on the journalistic sources consulted, to involve individuals who encountered the dogs in a work-related context. These included a farm worker responsible for the daily care of a guard dog (case 6), a domestic worker entering a private villa where large dogs had been left unconfined (case 14), a delivery worker attacked while delivering a package (case 28), and a property inspector who entered a residence during routine professional duties (case 29). These episodes could suggest that certain occupational groups, particularly workers who access private properties or interact with dogs in unfamiliar environments, may be exposed to specific risks. Similar patterns have been described internationally, with delivery personnel, domestic workers and technical service providers showing elevated bite incidence [34].

Our findings confirm a marked predominance of Molossian-type dogs in fatal attacks recorded in Italy between 2009 and 2025. In 22 of the 54 documented cases (41 percent), the attacking dogs belonged to this morphological group. This proportion closely mirrors the findings of the earlier study by Ciceroni and Gostinicchi (2009) [3], in which Molossers were involved in approximately 45 percent of fatal incidents recorded between 1984 and 2009.

However, a new and notable trend concerns bull-type terriers. In the 1984–2009 dataset, no fatal attacks were attributed to this subgroup. In contrast, our current analysis identifies the first bull-type Molosser fatality in 2017, followed by a steady rise in subsequent years that culminated in 15 fatal attacks involving these breeds. This increase suggests a growing prevalence of bull-type terriers in the Italian canine population, possibly driven by market trends, cultural preferences or an underestimation of the behavioral risks they may pose.

From an ethological standpoint, Molossers, particularly bull-type breeds, are characterized by behavioral traits that may increase the severity of aggressive incidents, including strong bite retention, low thresholds for arousal, minimal warning signals prior to attack and high resistance to pain or external deterrents [35,36].

These features do not imply inherent danger but do underscore the need for heightened awareness and careful management, particularly in households that include vulnerable individuals such as young children or frail adults. A further implication of our findings is that the exclusive focus of current Italian legislation on individual owner responsibility does not fully reflect the circumstances in which many fatal attacks occur. Several incidents in our dataset involved dogs that were being supervised not by the primary owner but by temporary caregivers, such as grandparents or other relatives. This pattern is consistent with international evidence showing that dog bites often occur in familiar domestic settings where supervision is shared among multiple household members or acquaintances [37]. Research on child–dog interactions also indicates that caregivers who are not the dog’s regular handlers may underestimate risk or misinterpret canine behavior, which can reduce the effectiveness of supervision [38]. These dynamics suggest that prevention strategies based solely on the behavior or competence of a single owner are insufficient. A more realistic framework should consider the broader social environment in which the dog is managed, including the variability in caregiver capacity and household organization.

When viewed through the official FCI classification system, the distribution of breeds involved in fatal attacks in our dataset aligns with three broad functional groups historically selected for guarding, protection, hunting or herding tasks [14]. Molossian-type breeds (FCI Group 2—Section 2) derive from lines developed for property defense and personal protection, and many display behavioral tendencies associated with high strength, determination, and reduced sensitivity to external stimuli. Terriers (FCI Group 3—Section 3) were historically developed for vermin control and for hunting underground against foxes, rats and badgers, reflecting their original functional role within this group [39]. Sheepdogs and Cattledogs (FCI Group 1) combine territorial vigilance with rapid reactivity to perceived threats, and several breeds within this group exhibit structured predatory motor patterns linked to their herding ancestry [40]. At the national level, recent FCI estimates indicate that FCI-registered pedigree dogs account for approximately 18% of the total dog population in Italy, confirming that pedigree animals represent only a minority of dogs living in the country [41]. The recurrent involvement of specific FCI groups in fatal attacks therefore likely reflects functional selection pressures rather than population size alone.

In light of this evidence, renewed consideration should be given to implementing mandatory training programs for owners of breeds with high offensive potential. Such measures were previously embedded in Italian legislation until 2009 [42]. As the ownership of dogs selectively bred for strength, endurance, and grip continues to expand, it is essential that handlers are fully informed of the associated responsibilities and risks.

Against this backdrop of individual responsibility, the observed dynamics should be interpreted within a broader perspective that includes national exposure patterns and comparative international evidence. The analysis of Italian cases shows that over 92% of fatal attacks involved owned dogs, more than half belonging to the victim, underscoring the limitations of a prevention model based solely on individual owner responsibility.

Only 7.4% of incidents involved stray dogs, confirming the effectiveness of the national stray control law (Framework Law 281/1991) [43]. This finding suggests that the current challenge lies not in the management of stray dogs but in preventing aggressive behavior among owned animals and establishing effective behavioral monitoring mechanisms. Notably, in several documented cases, the attacking dogs had a known history of previous aggression, yet no corrective or preventive measures were implemented (S2—Supplementary Materials). This observation further emphasizes the need for proactive behavioral monitoring and intervention protocols.

At the European level, an analysis of Eurostat data from 19 countries (1995–2016) revealed a consistent rise in dog-related fatalities, with the highest incidence among children and elderly individuals [44]. This trend situates the Italian experience within a broader context where shared demographic, social, and environmental factors contribute to increasing risk. Similarly, a multicenter Turkish study conducted between 2014 and 2023 found that more than one-third of animal-attack deaths were attributed to dogs, predominantly in rural settings and mainly involving male victims [45]. These findings indicate that in regions characterized by semi-owned dog populations and limited preventive policies, the likelihood of fatal attacks is amplified. In a global context, the United States represents a paradigmatic case for comparison. Between 2020 and 2023, an average of more than 55 fatal dog attacks were recorded annually in the United States, with pit bulls implicated in over 60 percent of multi-dog incidents [12]. DogsBite.org is not a scientific or peer-reviewed database; however, it remains one of the few publicly accessible repositories that systematically compile information on fatal dog attacks in the United States. Its value does not lie in scientific validation, but rather in illustrating how detailed, centralized reporting can support surveillance and policy discussion. By contrast, no comparable database exists in most European countries, including Italy, where the absence of a unified reporting system continues to limit the ability to monitor trends, compare cases, and develop evidence-based prevention strategies. Additional evidence from outside Europe and North America further supports the global consistency of the patterns observed in our dataset. In Ecuador, a recent One Health analysis of dog-bite incidents documented a predominance of child victims and highlighted substantial gaps in rabies vaccination coverage among owned dogs, illustrating how structural factors can amplify risk even in urban settings [46]. Comparable findings were reported in Peru, where a large community-based survey revealed significantly higher dog-bite rates in peri-urban areas with dense free-roaming dog populations and reduced access to healthcare, underscoring how socio-geographic inequities shape exposure dynamics [47]. Data from India similarly emphasize the high burden of dog-mediated rabies in contexts characterized by extensive free-roaming dog populations and fragmented preventive systems, reinforcing the importance of integrated surveillance and coordinated One Health policies [48]. Taken together, these findings demonstrate that the age-risk profiles, ownership patterns and environmental contexts observed in Italy align with well-documented trends across diverse low, middle and high-income regions. These international comparisons underscore the need for Italy to adopt similarly structured and evidence-based monitoring tools.

A further implication concerns the governance of behavioral risk data and the potential development of an integrated national surveillance system. In Italy, several regulatory instruments already require the recording and management of dog-related incidents, but these data streams remain fragmented across institutional actors. The current Ministerial Ordinance on the protection of public safety from dog aggression mandates the reporting of biting events to public veterinary services, the behavioral assessment of dogs involved in attacks, and the maintenance of a registry of high-risk dogs by local health authorities [6]. In parallel, the reform of the national animal health system introduced by the 2022 legislative decree reorganized veterinary information systems and strengthened interinstitutional data exchange, particularly in the context of zoonotic surveillance [49]. Hospital emergency departments also routinely record trauma mechanisms through standardized coding systems, including the ICD-10 category W54 (“bitten or struck by dog”), which is used in mortality statistics and in the external cause codes applied within regional hospital discharge flows. In 2024, the Ministry of Health formally requested all Regions to provide updated data on incidents involving dogs, acknowledging the current heterogeneity of reporting across territories [13].

Taken together, these sources demonstrate that the essential components for a coordinated surveillance framework already exist, although they currently operate in parallel and without interoperability. A national digital registry integrating veterinary notifications, behavioral risk assessments and hospital injury records, supported by pseudonymized identifiers, harmonized reporting pathways and a minimum dataset encompassing dog characteristics, ownership status, context of the event, severity of injury and prior aggression, would enable systematic epidemiological monitoring, facilitate forensic and behavioral analyses and strengthen public health decision making at both regional and national levels. In practical terms, a minimum variable set should include basic victim information (age, sex, role and circumstances), dog-related characteristics (type, size, ownership and supervision status, reproductive status and any prior episodes of aggression), situational elements (location, number of dogs involved, environmental context and proximate triggers), and clinical outcomes (type and severity of lesions, hospitalization and fatality), thereby ensuring data quality and interpretability across all domains of public health, forensic evaluation and behavioral risk assessment.

In light of these considerations, Italy would greatly benefit from establishing a national registry for monitoring dogs with behavioral risk, modeled on internationally validated systems. Such an instrument would harmonize data collection, facilitate early identification of high-risk subjects, and promote collaboration among veterinary medicine, forensic science and public health authorities. Within this framework, the role of veterinarians remains pivotal, as emphasized by the World Veterinary Association [50], which advocates a multidimensional approach grounded in clinical observation, responsible management and environmental awareness.

### Strengths and Limitations

This study represents the most comprehensive and methodologically rigorous epidemiological analysis of fatal dog attacks in Italy to date. By extending the observation window to 17 years and systematically collecting data from multiple verified journalistic sources, it fills a critical gap in national public health surveillance. The structured database enabled both descriptive and inferential statistical analysis, including multivariate modeling and interaction effects, which enhanced the depth and reliability of the findings.

The study also benefits from a multidisciplinary approach that integrates forensic, ethological and legal perspectives. Comparative analysis with U.S. data further contextualizes the Italian experience and underscores the relevance of centralized behavioral risk monitoring systems. The identification of recurring victim profiles, breed involvement and environmental contexts provides actionable insights for policymakers, veterinarians and public health authorities.

Despite its strengths, the study is subject to several limitations. First, the reliance on journalistic sources, while necessary due to the absence of a national registry, may introduce reporting bias, incomplete coverage or inaccuracies in breed identification and incident details. Although cross-verification and exclusion criteria were applied, the lack of access to forensic or veterinary records limits the ability to validate cases through institutional data.

Second, key variables such as the age, sex and behavioral history of the attacking dogs were often unavailable, precluding more granular analysis. The retrospective design also restricts direct observation of environmental or behavioral triggers, relying instead on secondary descriptions that may lack nuance.

Third, although statistical modeling was employed to explore associations and predictors, the absence of standardized national data constrained the use of more advanced techniques such as survival analysis or spatial epidemiology. The study does not account for near-fatal or non-lethal attacks, which may offer additional insights into risk dynamics and prevention strategies.

Finally, the difficulty in accessing detailed ICD-10 W54 mortality data in Italy, as recently acknowledged by the Ministry of Health, further limits the possibility of validating media-based findings through institutional sources [13]. These limitations reinforce the urgent need for a centralized and publicly accessible national database that systematically tracks fatal and non-fatal dog attacks, behavioral risk indicators and intervention outcomes.

## 5. Conclusions

This study provides the most comprehensive epidemiological profile of fatal dog attacks in Italy to date, spanning a 17-year period and revealing persistent patterns of vulnerability, breed involvement and environmental risk. Despite regulatory shifts toward owner accountability and behavioral monitoring, the number of fatalities has increased compared to the previous era of breed-specific legislation. The data underscore that fatal attacks are not random events but follow recurring dynamics, most notably involving molosser and bull-type breeds, owned dogs and vulnerable victims such as pre-school-aged children and the elderly.

The predominance of incidents in private settings, particularly within the victim’s household, highlights the limitations of current prevention strategies and the urgent need for structural reform. Comparative analysis with the United States demonstrates the value of centralized and publicly accessible databases in tracking behavioral risk factors and informing policy. Italy’s reliance on fragmented reporting and the absence of a national registry severely constrain its ability to monitor trends, intervene early and prevent future fatalities.

To address these gaps, we advocate for the creation of a national behavioral risk registry for dogs, integrated with veterinary, forensic and public health systems. Such a registry should include mandatory reporting of aggressive incidents, standardized behavioral assessments and enforceable intervention protocols. Only through coordinated and data-driven strategies can Italy move beyond reactive measures and toward a proactive model of canine aggression prevention, one that protects both human lives and animal welfare.

## Figures and Tables

**Table 1:** List of the 54 confirmed fatal dog attacks identified through the media-search procedure (2009–2025). Extended case-level information is provided in Supplementary Table S1, and full narrative reports with all primary links are available in Supplementary File S1.

Case	Year	Region	Main Verified News Source (Accessed on 2 December 2025)	Outcome Type
1	2009	Campania	Tgcom24—https://tinyurl.com/26jv2nr6	Death on scene
2	2009	Lazio	Il Giornale—https://tinyurl.com/yck23tsc	Death on scene
3	2009	Sicilia	La Stampa—https://tinyurl.com/4b5nyvb6	Death in hospital
4	2009	Sicilia	La Stampa—https://tinyurl.com/yhdkn3s5	Death on scene
5	2010	Puglia	La Gazzetta del mezzogiorno—https://tinyurl.com/nvb5srcd	Death in hospital
6	2010	Puglia	La Gazzetta del mezzogiorno—https://tinyurl.com/3n7mw3kh	Death on scene
7	2011	Puglia	La Repubblica—https://tinyurl.com/5bvcpczn	Death on scene
8	2011	Calabria	La Gazzetta del mezzogiorno—https://tinyurl.com/4ttmx4wf	Death in hospital
9	2011	Lombardia	Brescia today—https://tinyurl.com/35uja5nz	Death on scene
10	2012	Toscana	Il Tirreno—https://tinyurl.com/yabwukjx	Death on scene
11	2012	Lombardia	La Stampa—https://tinyurl.com/mreuwjv9	Death in hospital
12	2012	Sicilia	Il Corriere—https://tinyurl.com/9un8mvbb	Death in hospital
13	2012	Campania	Il Corriere del mezzogiorno—https://tinyurl.com/yftnbkjp	Death on scene
14	2012	Lazio	La Repubblica—https://tinyurl.com/yna29pta	Death in hospital
15	2013	Lombardia	La Repubblica—https://tinyurl.com/2fww2b9b	Death in hospital
16	2013	Toscana	Il Fatto Quotidiano—https://tinyurl.com/23m3vjcr	Death on scene
17	2013	Marche	Il Resto del Carlino—https://tinyurl.com/4f5bymcn	Death in hospital
18	2014	Lazio	Il Fatto Quotidiano—https://tinyurl.com/mt45w548	Death in hospital
19	2015	Friuli Venezia Giulia	Il Fatto Quotidiano—https://tinyurl.com/z5t5fmfy	Death in hospital
20	2015	Campania	La Repubblica—https://tinyurl.com/yws3vxv9	Death on scene
21	2016	Lombardia	La Repubblica—https://tinyurl.com/bddmb9hs	Death in hospital
22	2016	Sicilia	La Stampa—https://tinyurl.com/ywwzrbx5	Death on scene
23	2016	Abruzzo	La Stampa—https://tinyurl.com/3hka3b4s	Death in hospital
24	2016	Campania	Il Messaggero—https://tinyurl.com/3hkh69b2	Death on scene
25	2017	Puglia	La Repubblica—https://tinyurl.com/bdt6hh5p	Death on scene
26	2017	Lazio	Il Messaggero—https://tinyurl.com/2s3mbb84	Death on scene
27	2017	Lombardia	La Repubblica—https://tinyurl.com/5944xap6	Death on scene
28	2018	Sicilia	La Repubblica—https://tinyurl.com/84anpsxu	Death on scene
29	2018	Lazio	La Repubblica—https://tinyurl.com/yc2xrdaz	Death in hospital
30	2018	Puglia	La Repubblica—https://tinyurl.com/3ysf6dmm	Death in hospital
31	2019	Lazio	La Repubblica—https://tinyurl.com/ykrd49hb	Death on scene
32	2019	Friuli Venezia Giulia	La Repubblica—https://tinyurl.com/4efknuff	Death on scene
33	2019	Veneto	La Repubblica—https://tinyurl.com/yt93bfm9	Death on scene
34	2020	Lombardia	Il Giornale—https://tinyurl.com/yvy36cva	Death on scene
35	2020	Puglia	Il Messaggero—https://tinyurl.com/52cmh2tj	Death in hospital
36	2020	Piemonte	La Stampa—https://tinyurl.com/55pkhatu	Death on scene
37	2021	Lazio	Fanpage—https://tinyurl.com/58k2wh4r	Death on scene
38	2021	Calabria	La Stampa—https://tinyurl.com/3p4z36bv	Death on scene
39	2021	Piemonte	La Repubblica—https://tinyurl.com/2c2rxfca	Death on scene
40	2021	Emilia Romagna	La Stampa—https://tinyurl.com/4ys5uuct	Death on scene
41	2022	Puglia	L’Immediato—https://tinyurl.com/yj3eptbp	Unclear
42	2022	Emilia Romagna	La Stampa—https://tinyurl.com/4ays4394	Death in hospital
43	2023	Liguria	La Stampa—https://tinyurl.com/svr88pcx	Death in hospital
44	2023	Lombardia	Il Giorno—https://tinyurl.com/274nseym	Unclear
45	2024	Marche	Il Resto del Carlino—https://tinyurl.com/bdfdxrw5	Death on scene
46	2024	Lazio	La Stampa—https://tinyurl.com/3a5f84ww	Death on scene
47	2024	Campaniao	La Stampa—https://tinyurl.com/yc5yar49	Death on scene
48	2024	Piemonte	La Stampa—https://tinyurl.com/48ry42yz	Unclear
49	2024	Puglia	Tgcom24—https://tinyurl.com/4uhrayh3	Death in hospital
50	2025	Lazio	La Stampa—https://tinyurl.com/3wb99xs4	Death in hospital
51	2025	Lombardia	Il Corriere della Sera—https://tinyurl.com/f9r3yxz3	Death on scene
52	2025	Campania	La Stampa—https://tinyurl.com/52dyz9cr	Death on scene
53	2025	Sicilia	Il Corriere della Sera—https://tinyurl.com/2tdk8uh2	Death on scene
54	2025	Sicilia	La Stampa—https://tinyurl.com/yc4k892	Death on scene

**Table 2:** Summary of the primary trigger-related factors identified across 12 fatal dog attacks involving children aged 0–4 years.

Trigger/Dynamic Factor	Case IDs
Child on the ground	16, 27, 35
Child held in caregiver’s arms	22, 47
Child lying—sleeping	52
Child approaching the dog	18, 23, 27
Child’s position not reported	2, 5, 19, 48
Parent present	2, 5, 18, 22, 52
Relative present	16, 19, 23, 27, 48
Direct physical supervision (child held)	22, 47
Ineffective/distracted supervision	5, 16, 18, 19, 23, 27, 48, 52
Dog free/unrestrained	2, 5, 16, 19, 22, 27, 35, 47, 48, 52
Dog restrained (fenced or chained)	18, 23
Dogs escaping their enclosure	47
Multiple dogs present	5, 16, 27, 35, 47, 48
Attack in private garden/yard	5, 16, 18, 19, 23, 27, 35, 47
Attack in indoor environment	2, 48, 52

Note. The table reflects only those elements that were explicitly reported and could be consistently classified from journalistic sources.

## Data Availability

All data supporting the findings of this study are included within the article and its Supplementary Materials (Tables S1 and S2 and Supplementary File S1). Additional working spreadsheets used for descriptive and inferential statistical analyses, as well as preliminary graphical outputs prepared in Microsoft Excel, are available from the corresponding author upon reasonable request. No proprietary or confidential data were used or generated.

## Supplementary Materials

The following supporting information can be downloaded at: https://www.mdpi.com/article/10.3390/ani15243523/s1, Table S1: Overview of the 54 fatal dog attacks in Italy (2009–2025), including event, victim, and dog-related variables (date, location, environment, victim age/sex, number and breed of dogs, ownership status). Table S2: Data dictionary defining all variables included in the structured dataset, including variable names, descriptions, data types, and coding schemes. File S1: Chronological narrative reconstruction of each fatal event, with ethologically relevant contextual details, previous aggression history, and verified primary media sources.

## Abbreviations

The following abbreviations are used in this manuscript:

HE-V	Victim’s home environment
HE-NV	Non-victim home environment
PG-V	Victim’s private garden
PG-NV	Non-victim private garden
RUR	Rural environment
URB	Urban environment
PA	Pack attack

## References

[1] Hoffman J.M., O’Keefe K.A. (2022). Clinical and Epidemiologic Features of Persons Accessing Emergency Departments for Animal Bite Injuries in California, 2005–2019. J. Am. Vet. Med. Assoc..

[2] Giovannini E., Roccaro M., Peli A., Bianchini S., Bini C., Pelotti S., Fais P. (2023). Medico-Legal Implications of Dog Bite Injuries: A Systematic Review. Forensic Sci. Int..

[3] Ciceroni C., Gostinicchi S. (2009). Indagine Epidemiologica Sulle Aggressioni ad Esito Letale in Italia Negli Anni 1984–2009. Vet. Ital..

[4] Santoro V., Smaldone G., Lozito P., Smaldone M., Introna F. (2011). A Forensic Approach to Fatal Dog Attacks: A Case Study and Review of the Literature. Forensic Sci. Int..

[5] Ministero della Salute (2003). Ordinanza 9 Settembre 2003. Tutela dell’Incolumità Pubblica dal Rischio di Aggressioni da Parte di Cani Potenzialmente Pericolosi. Gazzetta Ufficiale della Repubblica Italiana.

[6] Ministero del Lavoro, della Salute e delle Politiche Sociali (2009). Ordinanza Concernente la Tutela dell’Incolumità Pubblica dall’Aggressione di Cani (Ordinanza 3 Marzo 2009). Gazzetta Ufficiale della Repubblica Italiana.

[7] Pomara C., D’Errico S., Jarussi V., Turillazzi E., Fineschi V. (2011). Cave Canem: Bite Mark Analysis in a Fatal Dog Pack Attack. Am. J. Forensic Med. Pathol..

[8] Giovannini E., Bianchini S., Roccaro M., Pelletti G., Grandis A., Peli A., Lenzi J., Pelotti S., Fais P. (2025). Morphometric Analysis of Dog Bitemarks: An Experimental Study. Forensic Sci. Int..

[9] Iarussi F., Cipolloni L., Bertozzi G., Sasso L., Ferrara M., Salerno M., Rubino G.T.R., Maglietta F., Dinisi A., Albano D. (2020). Dog-Bite-Related Attacks: A New Forensic Approach. Forensic Sci. Int..

[10] Benevento M., Trotta S., Iarussi F., Caterino C., Jarussi V., Solarino B. (2021). Multidisciplinary Analysis of Bite Marks in a Fatal Human Dog Attack: A Case Report. Leg. Med..

[11] Roccaro M., Grandis A., Peli A., Fais P., Pelotti S. (2021). Who Killed My Dog? Use of Forensic Genetics to Investigate an Enigmatic Case. Int. J. Leg. Med..

[12] DogsBite.org. U.S Dog Bite Fatalities (2020–2023): Annual Statistical Summary. https://www.dogsbite.org.

[13] Ministry of Health (2024). Nota per la Rendicontazione Regionale degli Episodi di Morsicatura/Aggression.

[14] Ministero della Salute Anagrafe degli Animali d’Affezione—Banca Dati Nazionale. https://www.salute.gov.it/anagcaninapublic_new.

[15] Legambiente Animali in Città: Ecco i Dati dell’Indagine di Legambiente. https://www.legambiente.it/comunicati-stampa/animali-in-citta-ecco-i-dati-dellindagine-di-legambiente/.

[16] Fédération Cynologique Internationale (2025). Breeds/Groups/Group 2—Pinscher & Schnauzer, Molossoid & Swiss Mountain and Cattle Dogs. Section 2: Molossian Type; Group 3 Section 3: Bull-Type Terriers; Group 1 Section 1: Sheepdogs. FCI Nomenclature—Breeds & Groups. Thuin (Belgium). https://www.fci.be/en/Nomenclature/Default.aspx.

[17] Chu A.Y., Ripple M.G., Allan C.H., Thogmartin J.R., Fowler D.R. (2006). Fatal Dog Maulings Associated with Infant Swings. J. Forensic Sci..

[18] Byard R.W. (2016). Domestic Dogs (*Canis lupus familiaris*) and Forensic Practice. Forensic Sci. Med. Pathol..

[19] Schalamon J., Ainoedhofer H., Singer G., Petnehazy T., Mayr J., Kiss K., Höllwarth M.E. (2006). Analysis of Dog Bites in Children Who Are Younger than 17 Years. Pediatrics.

[20] Frank D. (2013). Aggressive Dogs: What Questions Do We Need to Ask?. Can. Vet. J..

[21] Frank D., Lecomte S., Beauchamp G. (2021). Behavioral Evaluation of 65 Aggressive Dogs Following a Reported Bite Event. Can. Vet. J..

[22] McLennan T. (2023). Review of Literature on Interventions Aimed at Resolving Problems Caused by Predatory Behaviour in Dogs (*Canis familiaris*). Appl. Anim. Behav. Sci..

[23] Kouzos D., Katsos K., Zouzia E.I., Moraitis K., Vlachodimitropoulos D.G., Goutas N., Spiliopoulou C.A., Sakelliadis E.I. (2022). Non-Fatal Attacks by Dogs: Characteristics of Victims and Attacking Dogs, from the Forensic Perspective: A Series of 106 Cases From Athens, Greece, and Brief Review of the Literature. Cureus.

[24] Zoanetti J., Young J., Nielsen T.D. (2024). A Scoping Review of the Risks Posed by Companion Animals to Older Adults. Anthrozoös.

[25] Hammond A., Rowland T., Mills D.S., Pilot M. (2022). Comparison of behavioural tendencies between “dangerous dogs” and other domestic dog breeds: Evolutionary context and practical implications. Evol. Appl..

[26] Kleszcz A., Cholewińska P., Front G., Pacoń J., Bodkowski R., Janczak M., Dorobisz T. (2022). Review on Selected Aggression Causes and the Role of Neurocognitive Science in the Diagnosis. Animals.

[27] Notari L., Cannas S., Di Sotto Y.A., Palestrini C. (2020). A Retrospective Analysis of Dog–Dog and Dog–Human Cases of Aggression in Northern Italy. Animals.

[28] Borchelt P.L., Lockwood R., Beck A.M., Voith V.L. (1983). Attacks by Packs of Dogs Involving Predation on Human Beings. Public Health Rep..

[29] Fonseca G.M., Palacios R. (2013). An Unusual Case of Predation: Dog Pack or Cougar Attack?. J. Forensic Sci..

[30] Haug L.I. (2008). Canine Aggression Toward Unfamiliar People and Dogs. Vet. Clin. N. Am. Small Anim. Pract..

[31] Takáčová D., Skurková L., Mesárošová L., Lešková L., Kottferová L., Packová A., Vajányi D., Kottferová J. (2021). Dog Tethering in Slovakia: Legal, Ethical and Behavioral Aspects and Dog Welfare Implications. Animals.

[32] Marion M., Béata C., Sarcey G., Delfante S., Marlois N. (2018). Study of Aggressiveness in Livestock Guarding Dogs Based on Rearing Method. J. Vet. Behav..

[33] Cobb M.L., Branson N.J., McGreevy P.D., Bennett P. (2021). The Animal Welfare Science of Working Dogs: Current Knowledge and Research Priorities. Front. Vet. Sci..

[34] Owczarczak-Garstecka S.C., Christley R.M., Watkins F., Yang H., Westgarth C. (2021). “If You Don’t See the Dog, What Can You Do?” Using Procedures to Negotiate the Risk of Dog Bites in Occupational Contexts. Int. J. Environ. Res. Public Health.

[35] D’Ingeo S., Ferlisi G., Minunno M., Palmisano G.L., Ventriglia G., Siniscalchi M., Quaranta A. (2022). Motivations of Human Helping Behavior towards Dogs. Vet. Sci..

[36] D’Ingeo S., Iarussi F., De Monte V., Siniscalchi M., Minunno M., Quaranta A. (2021). Emotions and Dog Bites: Could Predatory Attacks Be Triggered by Emotional States?. Animals.

[37] Duncan-Sutherland N., Lissaman A.C., Shepherd M., Kool B. (2022). Systematic Review of Dog Bite Prevention Strategies. Inj. Prev..

[38] Arhant C., Landenberger R., Beetz A., Troxler J. (2016). Attitudes of Caregivers to Supervision of Child–Family Dog Interactions in Children up to 6 Years—An Exploratory Study. J. Vet. Behav..

[39] Stafford K. (2007). The Welfare of Dogs.

[40] Lord K., Schneider R.A., Coppinger R., Serpell J. (2016). Evolution of working dogs. The Domestic Dog: Its Evolution, Behavior and Interactions with People.

[41] Márton A. (2024). The Distribution of FCI Pedigree Dogs in the European Union in 2022. Fédération Cynologique Internationale (FCI) Report.

[42] Proposta di Legge Parlamentare. https://www.anmvioggi.it/images/IL_TESTO_DELLA_PLP_CON_EMENDAMENTI_APPROVATI.pdf.

[43] Parlamento Italiano (1991). Legge Quadro in Materia di Animali di Affezione e Prevenzione del Randagismo (Legge 14 Agosto 1991, n. 281). Gazzetta Ufficiale della Repubblica Italiana.

[44] Sarenbo S., Svensson P.A. (2021). Bitten or struck by dog: A rising number of fatalities in Europe, 1995–2016. Forensic Sci. Int..

[45] Atan T., Ersoy G., Çetin E., Kalkan E., Yildiz S. (2025). Deaths in Animal Attacks: A 1080-Year Retrospective Multicenter Study from Turkey (2014–2023). J. Forensic Sci..

[46] Calderón G.J., Poveda S., León Sosa A., Mora N., López Béjar M., Orlando S.A., García-Bereguiain M.A. (2023). Dog Bites as a Zoonotic Risk in Ecuador: Need for the Implementation of a One Health Approach. One Health.

[47] De la Puente-León M., Levy M.Z., Zegarra E., Paz-Soldán V.A., Bernedo R., Cornejo-Rosello I.F., Monroy Y. (2020). Spatial Inequality Hides the Burden of Dog Bites and the Risk of Dog-Mediated Human Rabies. Am. J. Trop. Med. Hyg..

[48] John D., Jha N., Kakkar M., Thakur J.S. (2021). Burden of Illness of Dog-Mediated Rabies in India: A Systematic Review and Meta-Analysis. One Health.

[49] Italian Government (2022). Legislative Decree 5 August 2022, No. 136. Implementation of Regulation (EU) 2016/429 on transmissible animal diseases and reorganization of national animal-health legislation. Gazzetta Ufficiale della Repubblica Italiana—Serie Generale.

[50] World Veterinary Association (2024). Position Statement on Control of Inappropriately Aggressive Dogs.

