# Supplementary material for: Fatal Dog Attacks in Italy (2009–2025): The Urgent Need for a National Risk Registry
Source: Animals (Basel). 2025 Dec 6;15(24):3523. doi: 10.3390/ani15243523 (PMC12729952; doi:10.3390/ani15243523)
Supplement: Supplementary file 1 [file animals-15-03523-s001.zip › Supplementary file S1.pdf]

CASE NARRATIVE

Known previous aggression → 11 cases (3, 4, 10, 14, 25, 27, 36, 39, 42, 47, 52)
Predatory attack (pursuit/child at play/runner) → 4 cases (3, 10, 12, 27)
Resource-guarding/meal-time context → 7 cases (1, 4, 18, 32, 34, 42, 54)

Case 1

Event Description

In January 2009, a 68-year-old woman with a physical disability was fatally attacked by a group of stray dogs in a rural area near Montesarchio, southern Italy. The victim, who lived alone, was found outside her home by a neighbor. Forensic examination revealed severe facial injuries, including traumatic avulsion of the nose and ears.

Context of the Attack

The woman reportedly fed local stray dogs, as food containers were discovered near her property.

Previous History

No documented episodes of aggression involving these dogs were reported.

Reference

https://www.tgcom24.mediaset.it/cronaca/articoli/437779/aggredita-da-cani-muore-un-anziana.shtml; https://www.tvsette.net/anziana-aggredita-e-uccisa-da-cani-randagi-nel-sannio/

Case 2

Event Description

In February 2009, an 11-month-old boy was fatally attacked by a Neapolitan Mastiff in the private garden of his home in Genzano di Roma, Italy. The infant was in a stroller when the family-owned dog assaulted him. The grandmother, who attempted to intervene, sustained multiple fractures.

Context of the Attack

The attack occurred within the family residence.

Previous History

The four-year-old dog was registered, vaccinated, and had no history of aggression.

Reference

https://www.ilgiornale.it/news/bambino-sbranato-cane-genzano-si-valuta-posizione-padre.html#google_vignette

---

Case 3

Event Description

In March 2009, a 10-year-old boy was fatally attacked by a pack of approximately twenty stray dogs in Sampieri, Sicily. He was knocked from his bicycle and mauled, sustaining fatal injuries to the head, neck, and chest.

Context of the Attack

The attack occurred while the boy was cycling in a coastal village.

Previous History

The same group of dogs had previously attacked a man and a 9-year-old child, and had bitten tourists the previous summer.

Reference

https://www.lastampa.it/cronaca/2009/03/15/news/bimbo-ucciso-da-un-branco-di-cani-1.37083922/

Case 4

Event Description
In July 2009, a 6-year-old boy was fatally attacked by dogs kept in an enclosure adjacent to his home in Acireale, Sicily. The child sustained a fatal bite to the jugular region. Evidence suggested the animals may have been trained for fighting.
Context of the Attack
The boy entered the enclosure through a gap in the fence.
Previous History
No formal aggression records existed, but the environment suggested an increased risk of dangerous behavior.
Reference
https://www.lastampa.it/cronaca/2009/07/22/news/il-fratello-della-vittima-di-acireale-1.37062518/; https://www.avvenire.it/attualita/pagine/acireale-bimbo-sbranato-i-cani-erano-del-fratello_200907230859137730000

Case 5
Event Description
In August 2009, a 2-year-old boy was fatally attacked by a Rottweiler on a family-owned farm near Foggia, Italy. The child sustained severe injuries to the torso and neck and died in hospital after several days.
Context of the Attack
The dog had been released from its enclosure, which had reportedly been left open by mistake.
Previous History
No prior aggression was reported for the dog.
Reference
https://www.ilsecoloxix.it/italia/2010/08/12/news/non-ce-l-ha-fatta-il-bimbo-azzannato-dal-cane-di-papa-1.33076572; https://www.lagazzettadelmezzogiorno.it/news/puglia/212612/foggia-muore-bimbo-azzannato-da-rottweiler.html
----

Case 6
Event Description
In December 2023, a 52-year-old Romanian farm worker was found dead near Borgo Mezzanone, Foggia. His body was decapitated, and forensic evidence suggested the involvement of a Rottweiler used to guard the property.
Context of the Attack
The victim was responsible for the dog's care and was the only worker able to handle the animal.
Previous History
The dog had no official aggression reports but was considered difficult to manage.
Reference
https://www.lagazzettadelmezzogiorno.it/news/puglia/243609/foggia-romeno-con-testa-mozzata-forse-staccata-a-morsi-da-cane-rottweiler.html; https://corrieredelmezzogiorno.corriere.it/napoli/notizie/cronaca/2010/27-dicembre-2010/rotweiller-stacca-testa-un-romenoipotesi-choc-aggredito-sbranato-vivo-181148155031.shtml
----

Case 7

Event Description
In 2011, an 84-year-old man was fatally attacked by two German Shepherds in Carovigno, Brindisi,

southern Italy. He was found on the roadside with multiple injuries and was pronounced dead at the scene.

Context of the Attack

The victim was walking toward his farmland when the attack occurred.

Previous History

The dogs had no microchips or identifying tags, and no prior aggression had been documented.

Reference

https://www.brindisireport.it/speciale/sbranato-da-un-branco-di-pastori-tedeschi-muore-pensionato-carovignese.html; https://bari.repubblica.it/cronaca/2011/03/26/news/anziano_sbranato_dai_cani_catturati_due_pastori_tedeschi-14115778/

Case 8

Event Description

In may 2011, a 74-year-old woman was fatally attacked by a group of approximately four dogs outside her home in Lattarico, Calabria, Italy. She sustained extensive injuries and died three days later in hospital.

Context of the Attack

The woman was attacked in front of her residence. A passerby witnessed the mauling and alerted emergency services.

Previous History

Five individuals were identified as owners of the dogs, but no previous aggression was reported.

Reference

https://www.lagazzettadelmezzogiorno.it/news/italia/274358/aggredita-da-cani-morta-in-ospedale.amp; https://www.quotidianodelsud.it/archivio/2011/05/09/lattarico-donna-sbranata-da-un-branco-di-cani-cinque-indagati

---

Case 9

Event Description

In 2011, a 73-year-old woman was fatally attacked by her German Shepherd in Carpenedolo, Brescia, northern Italy. The animal lunged at her throat, causing fatal exsanguination.

Context of the Attack

The attack occurred in the private courtyard of her home while the dog was chained.

Previous History

The dog had previously been described as calm, but in the days before the incident had been chained more frequently, possibly leading to stress.

Reference

https://www.fanpage.it/attualita/esce-in-cortile-e-viene-azzannata-dal-suo-cane-muore-una-73enne/; https://www.bresciatoday.it/cronaca/carpenedolo-morta-gola-dilaniata-dal-cane.html

---

Case 10

Event Description
On February 29, 2012, a 50-year-old truck driver was fatally attacked by a pack of at least seven dogs in an industrial yard between Livorno and Pisa. The man sustained extensive mutilation to his lower limbs.
Context of the Attack
The attack occurred while the man was preparing his truck in a semi-isolated logistics yard.
Previous History
The semi-feral dogs were irregularly fed by a local resident and had been reported as intimidating and aggressive in the area.
Reference
https://www.iltirreno.it/livorno/cronaca/2012/02/28/news/camionista-muore-sbranato-dai-cani-randagi-al-biscottino-1.3231924

Case 11

Event Description
On March 2, 2012, a 74-year-old man was fatally attacked by five to six stray dogs in Muggiano, Milan. He sustained severe wounds to his face, arms, and legs, and later died in hospital.
Context of the Attack
The victim was walking along a semi-rural street near cultivated fields when surrounded by the dogs.
Previous History
No documented prior aggression by these dogs was found.
Reference
https://milano.repubblica.it/cronaca/2012/03/03/news/milano_gravissimo_in_ospedale_74enne_aggredito_da_branco_di_cani-30839841/; https://www.milanotoday.it/zone/baggio/forze-armate/muore-gaetano-g-sbranato-cani-2-marzo-2012.html; https://www.lastampa.it/cronaca/2012/03/02/news/milano-sbranato-e-ucciso-dai-randagi-br-1.36490780/

Case 12

Event Description
In 2012, a 73-year-old man was fatally attacked by two Cane Corsos in Campobello di Licata, Sicily. The victim was bitten on the face, arms, and legs, leading to fatal hemorrhage.
Context of the Attack
The dogs escaped from a nearby property and pursued the victim to the entrance gate of his home.
Previous History
No prior aggression was documented for the dogs involved.
Reference
https://www.corriere.it/cronache/12_aprile_21/cani-sbranato-agrigento_9fe70ffe-8bf1-11e1-bdb0-bf9acf202da2.shtml; https://www.agrigentonotizie.it/cronaca/campobello-di-licata-ucciso-dai-cani-corso-vicino-profeta-condanna-appello-marzo-2019.html

---

Case 13

Event Description
On June 23, 2012, a 70-year-old man was fatally attacked by his Cane Corso in Mondragone, Campania. The victim's body showed extensive mutilation consistent with a violent assault.
Context of the Attack
The incident occurred inside the victim's private residence.
Previous History
No public reports of aggression were recorded prior to the attack.
Reference
https://corrieredelmezzogiorno.corriere.it/napoli/notizie/spettacoli/2012/23-giugno-2012/settantenne-sbranato-suo-cane-201734766074.shtml; https://www.sivempveneto.it/caserta-settantenne-sbranato-dal-suo-cane-carabinieri-lo-abbattano/

Case 14

Event Description
On July 16, 2012, a 74-year-old domestic worker was fatally attacked by two Rottweilers in the garden of a private villa in Giustiniana, Rome. Despite medical treatment, she died two months later.
Context of the Attack
The woman, who had keys to the property, entered while the dogs had been intentionally left unconfined as a security measure.
Previous History
The dogs had previously displayed territorial aggression, though no prior physical attacks were recorded.
Reference
https://roma.repubblica.it/cronaca/2012/07/16/news/aggredita_da_due_rottweiler_donna_in_fin_di_vita-39133565/; https://www.romatoday.it/cronaca/processo-rosario-russo-donna-morta-sbranata.html

---

Case 15

Event Description
In 2013, a 47-year-old mechanic was fatally attacked by a Dogue de Bordeaux in Sannazzaro de' Burgondi, Lombardy. The victim sustained severe bites to the neck and shoulder, resulting in fatal hemorrhage.
Context of the Attack
The man had been temporarily caring for the dog, which belonged to a friend. The attack occurred while he was feeding or checking on the animal.
Previous History
No prior aggression was reported. The dog had been described as playful and docile.
Reference
https://laprovinciapavese.gelocal.it/pavia/cronaca/2013/04/17/news/azzannato-dal-cane-muore-dissanguato-1.6899609;
https://milano.repubblica.it/cronaca/2013/04/16/news/sbranato_dal_suo_cane_nel_pavese_morto_vano_l_intervento_del_118-56787149/

Case 16
Event Description
On June 8, 2013, a 17-month-old boy, David Gammarota, was fatally attacked by at least two family-owned dogs (German Shepherds and a German Shepherd–Rottweiler mix) in a private villa near Siena, Tuscany. The child sustained a deep femoral wound leading to rapid exsanguination.
Context of the Attack
The child exited his bedroom undetected, descended to the ground floor, and accessed an area where the dogs were kept.
Previous History
No documented aggression was reported for the animals involved.
Reference
https://www.lanazione.it/siena/cronaca/2013/06/09/901751-siena-orrore-morto-bambino-cani-tragedia.shtml; https://www.ilfattoquotidiano.it/2013/06/08/bambino-di-17-mesi-muore-sbranato-da-due-cani-a-siena/620473/

Case 17
Event Description
On November 18, 2013, a 90-year-old woman, Lucia Quattrini, was fatally attacked by a Cane Corso on family property in Montecosaro Alta, Macerata. She sustained multiple injuries and died the following day in hospital.
Context of the Attack
The woman was either feeding cats or attempting to separate the dog from a kitten when the attack occurred.
Previous History
No prior aggression had been reported.
Reference
https://www.ilrestodelcarlino.it/civitanova_marche/cronaca/2013/11/19/984531-cane-uccide-donna-montecosaro.shtml; https://montecorriere.wordpress.com/2013/11/19/montecosaro-morta-la-donna-aggredita-dal-cane/; https://www.cronachemaceratesi.it/2013/11/21/azzannata-dal-cane-a-montecosaro-lultimo-saluto-a-lucia-quattrini/402138/

Case 18
Event Description
On September 24, 2014, a 3-year-old girl was fatally attacked by her family's German Shepherd in Fiano Romano, near Rome. She sustained multiple severe bites, including a fatal tracheal rupture.
Context of the Attack
The child exited the house and approached the dog enclosure while her father was bringing food.
Previous History
No history of aggression was documented.
Reference
https://www.ilfattoquotidiano.it/2014/09/25/roma-bambina-di-tre-anni-muore-azzannata-dal-suo-cane-nel-cortile-di-casa/1132715/; https://www.agi.it/cronaca/news/2014-09-25/tragedia_vicino_roma_azzannata_dal_suo_cane_muore_bimba_di_3_anni-10572/;

https://www.rainews.it/archivio-rainews/articoli/Roma-bambina-di-tre-anni-azzannata-e-uccisa-dal-suo-cane-93d2f77c-e09e-4fce-98cb-de274938790f.html

---

Case 19

Event Description

On May 25, 2015, a 2.5-year-old girl, Astrid Guarini, was fatally attacked by a Belgian Shepherd in the garden of her relatives' home in San Martino al Tagliamento, Pordenone. She died shortly after arrival at hospital.

Context of the Attack

The victim was playing in the garden with her cousin when attacked by her aunt's dog.

Previous History

The dog had no recorded aggression history, though warning signs at the property indicated trained guard dogs.

Reference

https://www.lastampa.it/cronaca/2015/05/25/news/azzannata-da-un-cane-in-giardino-bimba-di-tre-anni-muore-a-pordenone-1.35265670/; https://www.ilfattoquotidiano.it/2015/05/25/pordenone-bambina-di-due-anni-uccisa-dal-cane-di-una-parente/1717788/; https://www.rainews.it/archivio-rainews/articoli/Pordenone-Bimba-due-anni-azzannata-da-cane-morta-in-ospedale-ef4e86dd-987d-4836-8a61-31373662b749.html

---

Case 20

Event Description

On October 29, 2014, the body of a 61-year-old man, Vincenzo Zibella, was discovered in an olive grove near Capua, Campania. He had multiple canine bite injuries consistent with an attack by four Cane Corsos from a nearby property.

Context of the Attack

The victim was working alone harvesting olives when the suspected dogs escaped from adjacent land.

Previous History

No prior aggression had been officially recorded.

Reference

https://napoli.repubblica.it/cronaca/2015/10/24/news/uomo_trovato_morto_nel_casertano_sul_corpo_morsi_di_cani-125794578/; https://www.casertanews.it/cronaca/sbranato-cani-processo-curti.html; https://www.corrierece.it/notizie-cronaca/2015/10/25/choc-santangelo-in-formissequestrati-i.html

---

Case 21

Event Description

On November 4, 2014, a 76-year-old woman, Maria Rosa Pozzi, was fatally attacked by two American Staffordshire Terriers in the courtyard of her home in Inveruno, Milan province. Despite emergency surgery, she died within hours.

Context of the Attack

The woman was in the courtyard near the dogs' feeding area when attacked.

Previous History

No prior aggression reports existed.

Reference
https://guidominciotti.blog.ilsole24ore.com/2016/06/18/inveruno-milano-azzannata-a-morte-dai-cani-del-figlio-inutili-i-soccorsi/?refresh_ce=1; https://www.ilfattoquotidiano.it/2016/06/17/milano-morta-76enne-azzannata-alla-testa-da-2-suoi-cani-carabinieri-hanno-sparato-aria-per-liberarla/2839507/; https://milano.repubblica.it/cronaca/2016/06/17/news/azzannata_da_due_cani-142232906/

Case 22
Event Description
On August 17, 2015, an 18-month-old boy was fatally attacked by a family-owned Dogo Argentino in the garden of his home in Mascalucia, Catania province. He sustained fatal head and neck injuries.
Context of the Attack
The child was near a small inflatable pool in the garden when attacked. His mother attempted to intervene and was also injured.
Previous History
No aggression history had been documented for the two household dogs.
Reference
https://www.rainews.it/archivio-rainews/articoli/Tragedia-nel-catanese-bimbo-di-un-anno-e-mezzo-morto-sbranato-da-due-cani-1cd7ff7f-6af7-4786-9a40-168353481252.html;
https://www.lastampa.it/cronaca/2016/08/18/news/la-madre-del-bimbo-sbranato-dai-cani-lo-avevo-in-braccio-quando-uno-dei-cani-lo-ha-aggredito-1.34821396/;
https://tg24.sky.it/cronaca/2016/08/16/bambino-ucciso-da-cani-nel-catanese-mascalucia

---

Case 23
Event Description
On October 19, 2016, a 19-month-old boy, Ferdinando Di Rocco, was fatally attacked by a chained Cane Corso owned by his father in Cepagatti, Pescara province. The child sustained fatal cranial trauma.
Context of the Attack
The victim wandered unattended into the yard where the guard dog was tethered.
Previous History
No aggression history had been documented.
Reference
https://www.lastampa.it/cronaca/2016/10/21/news/bimbo-azzannato-da-un-cane-per-la-sua-morte-indagato-il-padre-con-la-moglie-1.34790661/; https://www.ilcentro.it/pescara/bimbo-di-19-mesi-ucciso-dal-cane-il-padre-condannato-a-un-anno-1.2465186; https://www.rainews.it/archivio-rainews/articoli/Azzannato-da-cane-muore-bimbo-di-due-anni-e-mezzo-544adf8b-8161-4716-a25a-d95a28dafaf9.html

---

Case 24
Event Description
On November 7, 2016, a 78-year-old man, Pasquale Malafronte, was fatally attacked by his two Cane Corsos in Battipaglia, Salerno province. He sustained a fatal carotid artery injury and extensive trauma.

Context of the Attack

The attack occurred at the victim's rural facility, possibly triggered by the presence of his small dog.

Previous History

No prior aggression had been reported.

Reference

https://www.ilmessaggero.it/primopiano/cronaca/sbranato_cani_pasquale_malafronte_battipaglia-2066580.html; https://www.salernotoday.it/cronaca/morto-battipaglia-pasquale-malafronte-cane-corso-7-novembre-2016.html

---

Case 25

Event Description

On April 18, 2017, 77-year-old Vito Zaccaria was fatally attacked in Francavilla Fontana, Brindisi province. He sustained deep lacerations and tissue avulsion consistent with a dog mauling. DNA evidence confirmed involvement of three dogs (two Pit Bulls and one mixed-breed).

Context of the Attack

The man was attacked while outside his home; the same dogs later attacked another person in the area.

Previous History

Yes — the same dogs attacked a 75-year-old woman six days later.

Reference

https://bari.repubblica.it/cronaca/2017/10/10/news/brindisi_l_esame_del_dna_incastra_i_cani_che_uccisero_un_anziano_denunciati_i_proprietari-177877639/; https://www.fanpage.it/attualita/ex-maresciallo-trovato-morto-e-seminudo-in-strada-forse-aggredito-dai-cani-dopo-un-malore/; https://www.brindisireport.it/cronaca/indagati-per-omicidio-colposo-proprietari-pitbull-che-sbranarono-pensionato-di-Francavilla-Fontana.html

---

Case 26

Event Description

An 88-year-old woman, Vincenza Mei, was fatally attacked by her recently adopted molossoid dog in Poggio Catino, Rieti. She sustained exsanguination from severe neck wounds.

Context of the Attack

The victim was found deceased in her home with the dog present.

Previous History

No prior aggression was documented.

Reference

https://www.ilmessaggero.it/rieti/rieti_donna_morta_sbranata-2550536.html; https://www.rietilife.com/2017/07/08/donna-muore-azzannata-dal-cane-tragedia-poggio-catino/

Case 27

Event Description

On September 17, 2017, a 14-month-old girl named Victoria was fatally attacked by two pit bull-type dogs in the courtyard of her home in Flero, Brescia. She sustained multiple fatal bites to the head and neck.

Context of the Attack
The child exited the house unnoticed and entered the courtyard where the dogs were kept.
Previous History
Yes — neighbors reported previous aggression and poor containment of the dogs.
Reference
https://milano.repubblica.it/cronaca/2017/09/18/news/brescia_bambina_uccisa_dai_cani_nonno_indagato-175846196/; https://guidominciotti.blog.ilsole24ore.com/2017/09/17/tragedia-a-brescia-bimba-di-un-anno-uccisa-dai-due-pitbull-di-casa-era-sola-con-il-nonno/

Case 28
Event Description
In 2017, a 55-year-old delivery worker was fatally attacked by three Cane Corsos at an agritourism facility near Noto, Siracusa. He sustained fatal injuries to his limbs.
Context of the Attack
The man was delivering a package when the dogs attacked upon his entry.
Previous History
Unknown; details of containment and prior behavior were not documented.
Reference
https://palermo.repubblica.it/cronaca/2018/01/05/news/siracusa_il_fattorino_aggredito_dai_cani_l_autopsia_conferma_e_morto_per_i_morsi-185883741/; https://www.fanpage.it/attualita/sbranato-a-morte-da-tre-cani-mentre-consegna-un-pacco-condannato-il-proprietario-dellanimale/

Case 29
Event Description
A 60-year-old man was fatally attacked by a German Shepherd while inspecting a villa in the province of Latina. He sustained critical injuries and later died in hospital.
Context of the Attack
The victim entered the property in the absence of the residents; the dog was unrestrained.
Previous History
Unknown.
Reference
https://roma.repubblica.it/cronaca/2018/09/10/news/fondi_medico_aggredito_e_ucciso_da_un_pastore_tedesco-206056577/; https://www.ilgiornale.it/news/cronache/latina-medico-aggredito-e-ucciso-cane-pastore-tedesco-1573931.html
----
Case 30
Event Description
A 62-year-old man was fatally attacked by two pit bull-type dogs while harvesting wild vegetables in Barletta-Andria-Trani province. He died in hospital after two days of critical care.
Context of the Attack
The man was in a rural field when attacked.

Previous History
Unknown. The dogs were microchipped and seized by authorities.
Reference
https://bari.repubblica.it/cronaca/2018/11/04/news/sbranato_vivo_da_due_pitbul_62enne_muore_a_san_ferdinando_di_puglia-210737958/;
https://www.lagazzettadelmezzogiorno.it/news/home/1076708/muore-62enne-sbranato-da-due-pitbull-mentre-va-in-bici-3-indagati.html
----
Case 31
Event Description
A 43-year-old man in Rome was fatally attacked by his Cane Corso during a routine walk in a park. He sustained severe lacerations to his right hand and arm, leading to massive blood loss and death from exsanguination.
Context of the Attack
The attack occurred during a normal afternoon walk. The precise trigger is unknown.
Previous History
No history of aggression had been reported.
Reference
https://roma.repubblica.it/cronaca/2019/03/24/news/tiago_sbranato_cane-222387720/;
https://www.ilmessaggero.it/roma/senzarete/cane_ucciso_padrone_recupero_ultime_notizie-8400964.html
----

Case 32
Event Description
A 74-year-old man was fatally attacked by two American Staffordshire Terriers while feeding them in a fenced enclosure at his sister-in-law's home in rural Pordenone. His wife sustained minor injuries while attempting to intervene.
Context of the Attack
The attack occurred during feeding inside the enclosure.
Previous History
No prior aggression was documented.
Reference
https://www.repubblica.it/cronaca/2019/10/25/news/aggredito_da_due_cani_che_lo_conoscevano_muore_74_enne-239480674/; https://www.corriere.it/cronache/19_ottobre_25/pordenone-74enne-muore-sbranato-cani-cognata-gravissima-moglie-ce1e2d48-f72e-11e9-9ad7-81cfe71b7fb2.shtml
----

Case 33
Event Description
In the province of Padua, a 49-year-old man was fatally attacked by two Rottweilers owned by his partner. He sustained catastrophic injuries to the head and upper limbs and died shortly after the assault.
Context of the Attack
The attack occurred within the partner's property; the exact trigger is unclear.

Previous History
No documented aggression.
Reference
https://www.repubblica.it/cronaca/2019/12/12/news/padova_uomo_sbranato_dai_suoi_rottweiler-243262340/; https://corrieredelveneto.corriere.it/padova/cronaca/19_dicembre_12/padova-ucciso-cani-rottweiler-fidanzata-0182592c-1cb7-11ea-8519-90a1403f658f.shtml
----

Case 34
Event Description
Leonarda Piga, a 64-year-old woman, was fatally attacked by two female Great Danes in Zeme, Lombardy. She was found in a pool of blood with multiple severe bite wounds and fractures.
Context of the Attack
The victim was caring for the animals at a dog breeding facility while the owner was absent. The incident likely occurred during feeding.
Previous History
No aggression previously reported.
Reference
https://www.ilgiornale.it/news/cronache/lomellina-64enne-sbranata-alani-e-trovata-senza-vita-1822702.html;
https://www.ilmessaggero.it/italia/donna_uccisa_cani_alani_oggi_pavia_5_febbraio_2020-5031491.html
----

Case 35
Event Description
An 8-month-old infant was fatally attacked by the family's Cane Corso in the garden of their home in Lecce province, Apulia. The child sustained head injuries and died shortly after admission to hospital.
Context of the Attack
The infant was with his mother in the garden when the attack occurred suddenly.
Previous History
No reports of aggression.
Reference
https://www.lecceprima.it/cronaca/bimbo-morto-morso-cane-tricase-11-aprile-2020.html;
https://www.ilmessaggero.it/italia/morto_bimbo_cane_tricase_funerale_ultime_notizie_13_aprile_2020-5168760.html
----

Case 36
Event Description
On December 18, 2020, Mariangela Zaffino, a 74-year-old woman, was fatally attacked by five Czechoslovakian Wolfdogs in her home in Grugliasco, Turin province. She died from exsanguination due to multiple bites.

Context of the Attack

The dogs were owned by her daughter, who housed five wolfdogs in a confined indoor environment. Neighbors had previously raised concerns.

Previous History

Yes — neighbors reported complaints about safety and problematic behavior.

Reference

https://www.lastampa.it/torino/2020/12/22/news/donna-sbranata-dai-cani-la-figlia-indagata-per-omicidio-colposo-1.39689624/; https://torino.corriere.it/notizie/cronaca/25_marzo_01/torino-anziana-sbranata-da-cinque-lupi-cecoslovacchi-condannata-la-figlia-0841765c-d695-4bf2-9eea-bafd8ebd0xlk.shtml

---

Case 37

Event Description

In April 2021, the body of 80-year-old Americo Tullio was found in a rural area between Morolo and Ferentino, Lazio. He had severe injuries consistent with dog bites. Two pit bull-type dogs belonging to a local resident were seized and linked to the attack through forensic DNA analysis.

Context of the Attack

The victim was foraging for wild asparagus when attacked.

Previous History

No prior aggression reported.

Reference

https://www.ciociariaoggi.it/news/cronaca/231285/anziano-sbranato-dai-pitbull-in-aula-sentito-anche-il-ris.html; https://www.fanpage.it/roma/esce-in-cerca-di-asparagi-tragica-fine-per-un-80enne-trovato-morto-con-il-corpo-dilaniato-dai-cani/

Case 38

Event Description

A 20-year-old woman was fatally attacked by a pack of twelve dogs, mostly Maremma Sheepdogs, while exploring a picnic area in Monte Fiorino, Catanzaro province. She sustained extensive injuries to her back and legs.

Context of the Attack

The dogs were likely guarding livestock.

Previous History

Not confirmed.

Reference

https://www.repubblica.it/cronaca/2021/08/26/news/ragazza_uccisa_da_cani_randagi_satriano-315439014/;https://www.lastampa.it/la-zampa/2021/08/28/news/catturato_uno_dei_cani_che_ha_sbranato_la_ragazza_in_calabria_il_sindaco_di_satriano_non_sono_randagi_ma_a_guardia_dei_g-367911119/

Case 39

Event Description
A 64-year-old woman in Verbano-Cusio-Ossola province was killed by her Staffordshire Bull Terrier. The dog inflicted fatal wounds to her throat and neck.
Context of the Attack
The attack occurred in the victim's home.
Previous History
Yes — the dog had previously bitten the woman's brother.
Reference
https://torino.repubblica.it/cronaca/2021/08/25/news/morsi_al_collo_nuca_e_gambe_donna_di_64_anni_muore_dopo_essere_stata_aggredita_dal_cane-315253766/; https://www.novaratoday.it/zone/verbano-cusio-ossola/pieve-vergonte-donna-trovata-morta-in-casa-e-stata-sbranata-dal-suo-cane.html

----

Case 40
Event Description
An 89-year-old woman in Modena province was fatally attacked by two American Staffordshire Terriers after she accidentally entered a neighbor's courtyard. She sustained catastrophic injuries and died at the scene.
Context of the Attack
The victim, in a confused state, entered the dogs' territory.
Previous History
No prior aggression documented.
Reference
https://www.lastampa.it/la-zampa/2021/10/14/news/anziana_89enne_sbranata_il_veterinario_non_dipende_dalla_razza_dei_due_cani_ma_dalla_scarsa_socializzazione-367914299/; https://corrieredibologna.corriere.it/bologna/cronaca/21_ottobre_15/donna-sbranata-cani-sassuolo-molossi-affidati-canile-non-riusciamo-piu-ad-accarezzarli-c070b294-2dad-11ec-9ca5-f4817997a20d.shtml

----

Case 41
Event Description
A 66-year-old homeless man was fatally attacked by two Pit Bulls in rural Foggia province. He was found dead with extensive bite injuries.
Context of the Attack
The man had sought shelter in the countryside when the attack occurred.
Previous History
No reports of prior aggression.
Reference
https://www.immediato.net/2022/06/19/tragedia-nel-foggiano-uomo-aggredito-e-ucciso-da-due-pitbull-a-cerignola-ce-anche-un-ferito/; https://www.foggiatoday.it/cronaca/uccisione-senzatetto-straniero-cerignola-morsi-pitbull.html

----

Case 42
Event Description
A 68-year-old woman in Modena province was fatally attacked by her two Rottweilers in her garden while feeding them. She sustained severe bites to the head and arm.
Context of the Attack
The attack occurred during feeding activity.
Previous History
Yes — the same dogs had previously attacked a gardener.
Reference
https://www.lastampa.it/cronaca/2022/12/25/news/anziana_muore_sbranata_dai_suoi_cani_nel_modenese-12432215/;
https://bologna.repubblica.it/cronaca/2022/12/27/news/concordia_donna_sbranata_cani_ausl_modena_veterinari_intervento-380927347/

Case 43
Event Description
A 53-year-old woman was fatally attacked by her brother's Rottweiler in Imperia province while caring for the animal during his absence. The victim suffered multiple severe wounds and died despite medical intervention.
Context of the Attack
The attack occurred in the courtyard of the dog's home.
Previous History
No prior aggression reported.
Reference
https://www.lastampa.it/cronaca/2023/04/06/news/bordighera_muore_sbranata_rottweiler-12740200/; https://www.corriere.it/cronache/23_aprile_06/imperia-patrizia-marca-morta-sbranata-rottweiler-fratello-96487cae-d45a-11ed-b15d-00361b7c9d2d.shtml
----

Case 44
Event Description
An 86-year-old woman was fatally attacked by a Pit Bull in a public square in Pavia province. The dog escaped from its owner's control and bit her on the legs and throat.
Context of the Attack
The victim was walking in a public area when attacked.
Previous History
No prior aggression documented.
Reference
https://www.ilgiorno.it/pavia/cronaca/sbranata-pttbull-b16cfdec; https://www.ilfattoquotidiano.it/in-edicola/articoli/2023/10/22/pavia-muore-a-86-anni-sbranata-da-un-pitbull/7330497/

Case 45

Event Description
An 80-year-old woman in Pesaro Urbino province was fatally attacked by her Maremma Shepherd in the garden of her home. She sustained fatal neck injuries.
Context of the Attack
The attack occurred in the family garden; her husband attempted to intervene unsuccessfully.
Previous History
No prior aggression reported.
Reference
https://www.ilfattoquotidiano.it/in-edicola/articoli/2023/10/22/pavia-muore-a-86-anni-sbranata-da-un-pitbull/7330497/; https://www.ilrestodelcarlino.it/pesaro/cronaca/donna-morta-sbranata-dal-suo-cane-xxe0u121

Case 46
Event Description
A 39-year-old man was fatally attacked by three Rottweilers while jogging in the Manziana forest, Rome province. He sustained severe facial and arm injuries and died at the scene.
Context of the Attack
The dogs had escaped from a nearby home adjacent to the forest.
Previous History
No documented aggression.
Reference
https://www.lastampa.it/la-zampa/2024/02/11/news/roma_muore_sbranato_cane_rottweiler_jogging-422108641/; https://tg24.sky.it/roma/2024/02/11/muore-sbranato-rotweiller-manziana-roma
----

Case 47
Event Description
A 15-month-old child was fatally attacked by two Pit Bulls owned by neighbors. The child was in his uncle’s arms when the dogs attacked, pulling him down and inflicting fatal injuries.
Context of the Attack
The attack occurred in a residential area; family members tried to intervene with sticks.
Previous History
Yes — the same dogs had previously killed another dog.
Reference
https://www.lastampa.it/la-zampa/2024/04/22/news/eboli_bambino_ucciso_cani_pit_bull-422699256/; https://salerno.corriere.it/notizie/cronaca/24_aprile_24/eboli-francesco-pio-sbramato-dai-pitbull-indagati-la-madre-gli-zii-e-i-proprietari-dei-cani-5b71f22e-1a8a-4ed5-9c23-ba243bbcbxlk.shtml
----

Case 48

Event Description
A 5-month-old infant in Vercelli province was fatally attacked by a family-owned Pit Bull while being held by the grandmother in the courtyard.
Context of the Attack
The attack occurred in the family home environment.
Previous History
Not specified.
Reference
https://www.lastampa.it/vercelli/2024/05/18/news/bebe_sbranato_dal_pitbull_a_palazzolo_la_procura_di_vercelli_apre_uninchiesta-14313564/; https://torino.corriere.it/notizie/cronaca/24_maggio_18/bimbo-ucciso-dal-pitbull-a-palazzolo-vercellese-la-donna-urlava-salvate-michele-con-due-mattoni-e-una-scopa-abbiamo-allontanato-il-cane-4025e274-96ac-4ab5-bc5e-9be86b65dxlk.shtml
----

Case 49
Event Description
A 97-year-old woman in Lecce province was fatally attacked by the family's Cane Corso while performing household tasks. She sustained severe facial and head injuries and later died in hospital.
Context of the Attack
The incident occurred inside the family home. Police fatally shot the dog to stop the attack.
Previous History
No prior aggression documented.
Reference
https://www.tgcom24.mediaset.it/cronaca/puglia/lecce-morta-97enne-sbranata-da-cane-nipote_83397667-202402k.shtml; https://www.lecceprima.it/cronaca/aggressione-cane-corso-donna-alessano-6-giugno-2024.html
----

Case 50
Event Description
A 29-year-old woman was fatally attacked by five stray mixed-breed dogs in an abandoned house on the outskirts of Latina. She sustained fatal injuries and was found in critical condition.
Context of the Attack
The dogs lived in poor conditions and were neglected and malnourished.
Previous History
Yes — the same group of dogs had attacked another person.
Reference
https://roma.corriere.it/notizie/cronaca/25_luglio_19/sbranata-da-una-muta-di-cani-a-latina-le-foto-del-corpo-di-patricia-masithela-rubate-e-pubblicate-in-chat-su-whatsapp-f2ab10b7-2a1b-4fda-be35-a1b9faf3fxlk.shtml; https://www.ilfattoquotidiano.it/2025/07/21/patricia-masithela-foto-cadavere-whatsapp-notizie/8069054/

Case 51

Event Description
A 72-year-old man, a veterinarian and dog breeder, was fatally attacked by his own Great Danes in the garden of his home in Milan province. He sustained fatal head and neck injuries.
Context of the Attack
The attack occurred in the victim's private property.
Previous History
No aggression history reported.
Reference
https://milano.corriere.it/notizie/cronaca/25_gennaio_19/milano-veterinario-72enne-trovato-in-casa-senza-vita-potrebbe-essere-stato-sbranato-dai-suoi-cinque-alani-33476269-1a34-4085-b2fc-7f33c4f6fxlk.shtml; https://www.rainews.it/articoli/2025/01/veterinario-di-72-anni-trovato-morto-in-giardino-forse-ucciso-dai-suoi-alani-cerromaggiore-milano-9cd3f43b-b7ff-4a1d-b32c-8969cab70f1c.html
----

Case 52
Event Description
A 9-month-old girl was fatally attacked by the family's Pit Bull in Naples province. The father had fallen asleep with the child on the bed and awoke to find her fatally mauled.
Context of the Attack
The attack occurred in a domestic bedroom environment.
Previous History
Yes — the Pit Bull had previously killed another dog.
Reference
https://www.lastampa.it/cronaca/2025/02/18/news/omicidio_pitbull_bambina_hashish_padre-15009712/; https://www.rainews.it/articoli/2025/02/-acerra-bimba-sbranata-dal-pitbull-sequestrato-il-cellulare-del-papa-i-misteri-da-sciogliere-napoli-983f443c-2ad3-496a-b537-7df1ba4617e4.html
----

Case 53
Event Description
An 84-year-old man was fatally attacked by two Cane Corsos in Palermo province. He sustained extensive injuries to his arms and legs and died shortly after.
Context of the Attack
The dogs were owned by a neighbor and were found outside the owner's property.
Previous History
Not specified.
Reference
https://www.corriere.it/cronache/25_febbraio_17/palermo-nuovo-caso-di-aggressione-anziano-azzannato-e-ucciso-da-un-branco-di-cani-75cd7038-0316-439b-ba56-3b83f22a0xlk.shtml; https://www.lastampa.it/cronaca/2025/02/17/news/bagheria_uomo_aggredito_cani_muore-15007835/

Case 54

Event Description
A 62-year-old woman in Trapani province was fatally attacked by her mixed-breed dog while feeding it in the garage. She sustained fatal head and arm injuries.
Context of the Attack
The dog was chained at the time of the incident.
Previous History
No prior aggression reported.
Reference
https://www.lastampa.it/cronaca/2025/03/22/news/azzannata_uccisa_da_cane_donna_petrosino_trapani-15066819/; https://www.rainews.it/articoli/2025/03/muore-a-62-anni-sbranata-dal-cane-la-scoperta-del-marito-e-dal-figlio-72535511-02d4-45a4-a76c-ab39c2e67861.html
