# Supplementary material for: Fatal Dog Attacks in Italy (2009–2025): The Urgent Need for a National Risk Registry
Source: Animals (Basel). 2025 Dec 6;15(24):3523. doi: 10.3390/ani15243523 (PMC12729952; doi:10.3390/ani15243523)
Supplement: Supplementary file 1 [file animals-15-03523-s001.zip › Table S1.pdf]

Table S1. Overview of the variables considered (year and month of the incident, province in which the attack occurred, number of victims, their age and sex, total number of dogs involved, breed, ownership status, and the environment in which the event took place).

EVENT INFORMATION				VICTIM INFORMATION			DOG INFORMATION		
Case	***Date***	***Region***	***Environment***	***No.***	***Age***	***Sex***	***No.***	***Breed***	***Ownership***
1	01/2009	Campania	RUR	1	68y	F	Pa	Mixed breed	Stray
2	02/2009	Lazio	PG-V	1	11mo	M	1	Italian Mastiff	By Victim
3	03/2009	Sicilia	RUR	1	10y	M	Pa	Mixed breed	Stray
4	07/2009	Sicilia	PG-V	1	6y	M	Pa	Mixed breed/Molosser	By Victim
5	08/2010	Puglia	PG-V	1	2y	M	1	Rottweiler	By Victim
6	12/2010	Puglia	PG-NV	1	52y	M	1	Rottweiler	by third party
7	03/2011	Puglia	RUR	1	84y	M	2	German Shepherd	by third party
8	05/2011	Calabria	URB	1	74y	F	Pa	Mixed breed	by third party
9	11/2011	Lombardia	PG-V	1	73y	F	1	German Shepherd	By Victim
10	01/2012	Toscana	URB	1	50y	M	Pa	Mixed breed	Stray
11	03/2012	Lombardia	URB	1	74y	M	Pa	Mixed breed	Stray
12	04/2012	Sicilia	RUR	1	73y	M	2	Cane corso	by third party
13	06/2012	Campania	HE-V	1	70y	M	1	Cane corso	By Victim
14	09/2012	Lazio	PG-NV	1	74y	F	2	Rottweiler	by third party
15	04/2013	Lombardia	PG-V	1	47y	M	1	Dogue de Bordeaux	By Victim
16	06/2013	Toscana	HE-V	1	17 mo	M	2	German Shepherd	By Victim
17	11/2013	Marche	PG-V	1	90y	F	1	Cane Corso	By Victim
18	09/2014	Lazio	PG-V	1	3y	F	1	German Shepherd	By Victim
19	05/2015	Friuli Venezia G.	PG-V	1	2y	F	1	Belgian Shepherd	By Victim
20	10/2015	Campania	RUR	1	61y	M	Pa	Cane Corso	by third party
21	06/2016	Lombardia	PG-V	1	76 y	F	2	Amstaff	By Victim
22	08/2016	Sicilia	PG-V	1	18 mo	M	1	Dogo Argentino	By Victim
23	10/2016	Abruzzo	PG-V	1	19 mo	M	1	Cane Corso	By Victim
24	11/2016	Campania	RUR	1	78 y	M	2	Cane Corso	By Victim
25	04/2017	Puglia	RUR	1	77 y	M	Pa	2 Pit-bull-1M-breed dog	by third party
26	06/2017	Lazio	HE-V	1	88y	F	1	Pit bull	by victim
27	09/2017	Lombardia	PG-V.	1	13 mo	F	2	Pit-bull	by victim
28	01/2018	Sicilia	PG-NV	1	55 y	M	Pa	Cane Corso	by third party
29	09/2018	Lazio	HE-NV	1	60 y	M	1	German Shepherd	by third party
30	10/2018	Puglia	RUR	1	62 y	M	2	Pit-bull	by third party
31	03/2019	Lazio	URB	1	43 y	M	1	Cane Corso	by victim
32	10/2019	Friuli Venezia G.	HE-NV	1	74 y	M	2	Am. St. Terrier	by third party
33	12/2019	Veneto	PG-V	1	49 y	M	2	Rottweiler	by victim
34	02/2020	Lombardia	PG-NV	1	64 y	F	2	Great Danes	by third party
35	04/2020	Puglia	PG-V	1	8 mo	M	1	Cane Corso	by victim
36	12/2020	Piemonte	HE-V	1	74 y	F	Pa	Cz. Wolfdogs	by victim
37	04/2021	Lazio	RUR	1	80 y	M	2	Pit-bull	by third party
38	08/2021	Calabria	RUR	1	20 y	F	Pa	Maremma Sheepdog	by third party
39	08/2021	Piemonte	HE-V	1	64 y	F	1	Staff. bull terrier	by victim
40	10/2021	Emilia Romagna	PG-NV	1	89 y	F	2	Amstaff	by third party
41	06/2022	Puglia	RUR	1	66 y	M	2	Pit-bull	by third party
42	12/2022	Emilia Romagna	PG-V	1	68 y	F	2	Rottweiler	by victim
43	04/2023	Liguria	HE-NV	1	53 y	F	1	Rottweiler	by third party
44	10/2023	Lombardia	URB	1	86 y	F	1	Pit-bull	by third party
45	02/2024	Marche	PG- V.	1	80 y	F	1	Maremma Sheepdog	by victim
46	02/2024	Lazio	URB	1	39 y	M	Pa	Rottweiler	by third party
47	04/2024	Campania	PG-V.	1	15 mo	M	2	Pit-bull	by third party
48	05/2024	Piemonte	PG-V	1	5 mo	M	1	Pit-bull	by victim
49	06/2024	Puglia	HE-V	1	97 y	F	1	Cane Corso	by victim
50	01/2025	Lazio	PG-NV	1	29 y	F	Pa	Mixed-breed dog	by third party
51	01/2025	Lombardia	PG-V	1	72 y	M	2	Great Dane	by victim
52	02/2025	Campania	HE-V	1	9 mo	F	1	Pit-bull	by victim
53	02/2025	Sicilia	RUR	1	85 y	M	2	Cane Corso	by third party
54	03/2025	Sicilia	HE-V	1	62 y	F	1	Mixed-breed dog	by victim

(Legend: y = years; mo = months; HE-V = Victim's home environment; HE-NV = Non-victim home environment; PG-V = Victim's private garden; PG-NV = Non-victim private garden; RUR = Rural environment; URB = Urban environment); PA= Pack attack
