# Supplementary material for: Fatal Dog Attacks in Italy (2009–2025): The Urgent Need for a National Risk Registry
Source: Animals (Basel). 2025 Dec 6;15(24):3523. doi: 10.3390/ani15243523 (PMC12729952; doi:10.3390/ani15243523)
Supplement: Supplementary file 1 [file animals-15-03523-s001.zip › Table S2.pdf]

Table S2: Data dictionary for all variables included in the dataset.

VARIABLE NAME	*DESCRIPTION*	*TYPE*	*CATEGORIES / NOTES*
Case	Progressive case identifier	Integer	1–54
Date	Month and year of the fatal attack	Date	Format: mm/yyyy
Region	Italian region where the event occurred	Categorical	20 standard Italian regions
Environment	Setting in which the event occurred	Categorical	URB = urban; RUR = rural; PG-V = private garden (victim-owned); PG-NV = private garden (not victim-owned); HE-V = home environment (victim-owned); HE-NV = home environment (not victim-owned)
Victim_number	Number of victims involved in the event	Integer	Always 1 in this dataset
Victim_age	Age of the victim	Numeric	In years or months (Infants 0-12)
Victim_sex	Sex of the victim	Categorical	M / F
Dog_number	Total number of dogs involved	Integer	1, 2, or >2
Dog_breed	Breed or type of dog(s) involved	Text	As reported; FCI classification when applicable
Ownership_status	Ownership category of the dog(s)	Categorical	Victim-owned; third-party; stray/semi-owned
Pack_attack	Whether more than one dog was involved	Binary	Yes / No
Outcome	Location where death occurred	Categorical	On-scene / In-hospital
Context_description	Brief description of the attack circumstances	Text	Derived from media reports
